# Evaluation of GRCh38 and de novo haploid genome assemblies demonstrates the enduring quality of the reference assembly

**DOI:** 10.1101/gr.213611.116

**Published:** 2017-05

**Authors:** Valerie A. Schneider, Tina Graves-Lindsay, Kerstin Howe, Nathan Bouk, Hsiu-Chuan Chen, Paul A. Kitts, Terence D. Murphy, Kim D. Pruitt, Françoise Thibaud-Nissen, Derek Albracht, Robert S. Fulton, Milinn Kremitzki, Vincent Magrini, Chris Markovic, Sean McGrath, Karyn Meltz Steinberg, Kate Auger, William Chow, Joanna Collins, Glenn Harden, Timothy Hubbard, Sarah Pelan, Jared T. Simpson, Glen Threadgold, James Torrance, Jonathan M. Wood, Laura Clarke, Sergey Koren, Matthew Boitano, Paul Peluso, Heng Li, Chen-Shan Chin, Adam M. Phillippy, Richard Durbin, Richard K. Wilson, Paul Flicek, Evan E. Eichler, Deanna M. Church

**Affiliations:** 1 National Center for Biotechnology Information, National Library of Medicine, National Institutes of Health, Bethesda, Maryland 20894, USA;; 2 McDonnell Genome Institute at Washington University, St. Louis, Missouri 63018, USA;; 3 Wellcome Trust Sanger Institute, Wellcome Genome Campus, Hinxton, Cambridge CB10 1SA, United Kingdom;; 4 European Molecular Biology Laboratory, European Bioinformatics Institute, Wellcome Genome Campus, Hinxton, Cambridge CB10 1SD, United Kingdom;; 5 National Human Genome Research Institute, National Institutes of Health, Bethesda, Maryland 20892, USA;; 6 Pacific Biosciences, Menlo Park, California 94025, USA;; 7 Broad Institute, Cambridge, Massachusetts 02142, USA;; 8 Department of Genome Sciences, University of Washington School of Medicine, Seattle, Washington 98195, USA;; 9 Howard Hughes Medical Institute, University of Washington, Seattle, Washington 98195, USA

## Abstract

The human reference genome assembly plays a central role in nearly all aspects of today's basic and clinical research. GRCh38 is the first coordinate-changing assembly update since 2009; it reflects the resolution of roughly 1000 issues and encompasses modifications ranging from thousands of single base changes to megabase-scale path reorganizations, gap closures, and localization of previously orphaned sequences. We developed a new approach to sequence generation for targeted base updates and used data from new genome mapping technologies and single haplotype resources to identify and resolve larger assembly issues. For the first time, the reference assembly contains sequence-based representations for the centromeres. We also expanded the number of alternate loci to create a reference that provides a more robust representation of human population variation. We demonstrate that the updates render the reference an improved annotation substrate, alter read alignments in unchanged regions, and impact variant interpretation at clinically relevant loci. We additionally evaluated a collection of new de novo long-read haploid assemblies and conclude that although the new assemblies compare favorably to the reference with respect to continuity, error rate, and gene completeness, the reference still provides the best representation for complex genomic regions and coding sequences. We assert that the collected updates in GRCh38 make the newer assembly a more robust substrate for comprehensive analyses that will promote our understanding of human biology and advance our efforts to improve health.

The human reference genome assembly remains a critical resource for the biological and clinical research communities (International Human Genome Sequencing Consortium 2001, 2004). It is distinguished from the growing number of human genome assemblies in public databases by virtue of its long contig and scaffold N50s, high base-pair accuracy, and robust representations of repetitive and segmentally duplicated genomic regions, all of which reflect the clone-based assembly approach and Sanger sequencing methods that were the basis of its generation. In particular, it was the use of large insert BAC clones (>150 kb inserts) and the deep coverage provided by multiple end-sequenced clone libraries, coupled with extensive use of radiation hybrid, genetic linkage, and fingerprint maps, that made it possible to span large repetitive regions and achieve the as-yet unsurpassed contiguity of the reference. Assembled from the DNA of multiple donors, the reference was intended to provide representation for the pan-human genome, rather than a single individual or population group, and is a mosaic of haplotypes whose borders coincide with the underlying clone boundaries.

A revision to the assembly model, first used in the previous version of the reference, GRCh37 (GCA_000001405.1), expanded the ability of the reference assembly to represent the extent of structural variation and population genomic diversity whose discovery it facilitated (The International HapMap Consortium 2005; Kidd et al. 2008; Sudmant et al. 2010; Church et al. 2011; The 1000 Genomes Project Consortium 2015). The introduction of alternate loci scaffolds enabled GRCh37 to include additional sequence representations for the highly variant MHC region, as well as the divergent haplotypes of the *MAPT* and *UGT2B* loci, while retaining the linear chromosome representations familiar and intuitive to most users (Horton et al. 2008; Xue et al. 2008; Zody et al. 2008). A second feature of the updated model, assembly patches, permitted subsequent corrections and addition of new sequence representations to the GRCh37 assembly without changing the chromosome sequences or coordinates on which an increasing volume of data were being mapped (Zook et al. 2014; The 1000 Genomes Project Consortium 2015; Pierson et al. 2015). The assembly model remains for GRCh38, the current reference version. Together, these features of the assembly model helped ensure that the human reference assembly would continue to present the most accurate representation of the human genome possible while providing a stable substrate for large-scale analysis.

The GRCh37 assembly underwent 13 patch releases in the period from 2009 to 2013 (GCA_000001405.2–GCA_00000 1405.14). Despite the availability of these sequences in public databases, their use has been limited by the inability of common bioinformatics file formats and tool chains to manage the allelic duplication they introduce, as well as by their constrained representation in popular genome browsers (Church et al. 2015). In addition, the patches represented only a subset of the assembly updates made by the Genome Reference Consortium (GRC). Thus, coordinate changing assembly updates remain essential for users to access the full suite of assembly improvements, despite the challenge of transporting data and results to the new assembly (Hickey et al. 2013; Zhao et al. 2014).

In producing GRCh38, we of the GRC placed special emphasis on addressing the following types of assembly issues found in GRCh37: (1) resolution of tiling path errors and gaps associated with complex haplotypes and segmental duplications; (2) base-pair–level updates for sequencing errors; (3) addition of “missing” sequences, with an emphasis on paralogous sequences and population variation; and (4) providing sequence representation for genomic features, such as centromeres and telomeres. Making these updates involved the use of bioinformatics and experimental resources and techniques not previously available. We will demonstrate how the new approaches used in this effort result in a human reference genome assembly that is more contiguous and complete than ever before and that provides better gene and variant representation than GRCh37, features critical to both basic research and clinical uses of the assembly. We will also show how assembly updates in GRCh38 impact analyses throughout the genome, even in regions that are unchanged between the two assemblies. Together, these analyses suggest adoption of the new assembly will have a positive impact on both genome-wide analysis as well as regional analysis.

With long-range sequencing and assembly technologies making the generation of highly contiguous whole-genome de novo assemblies possible, the overall value of GRCh38 and the human reference genome assembly in general, must now also be considered (Chaisson et al. 2015b). The reference assembly is not just a substrate for alignment, but is also the coordinate system on which we annotate our biological knowledge. Several recently published individual human de novo assemblies have been favorably compared to GRCh38 with respect to continuity metrics, and although they each contain sequence not present in the reference assembly, none yet surpass the global quality of GRCh38 (Li et al. 2010; Steinberg et al. 2014; Berlin et al. 2015; Cao et al. 2015; Pendleton et al. 2015; Seo et al. 2016; Shi et al. 2016). Such assemblies are often suggested as sequence sources for use in closure of reference assembly gaps, whereas other studies have called for one or more individual genomes to replace the reference (Rosenfeld et al. 2012). To address these issues, we generated and evaluated a collection of de novo assemblies representing the essentially haploid complete hydatidiform mole samples CHM1 and CHM13 (Fan et al. 2002; Steinberg et al. 2014). The assemblies were derived from the same sequence data, but assembled using different algorithms and/or parameters, and assessed with a range of assembly metrics with respect to each other and GRCh38. To our knowledge, these efforts represent the first such assessment performed specifically to explore the suitability of de novo assemblies for use in curation or replacement of the human reference assembly.

## Results

### Assembly updates

**Figure 1.**
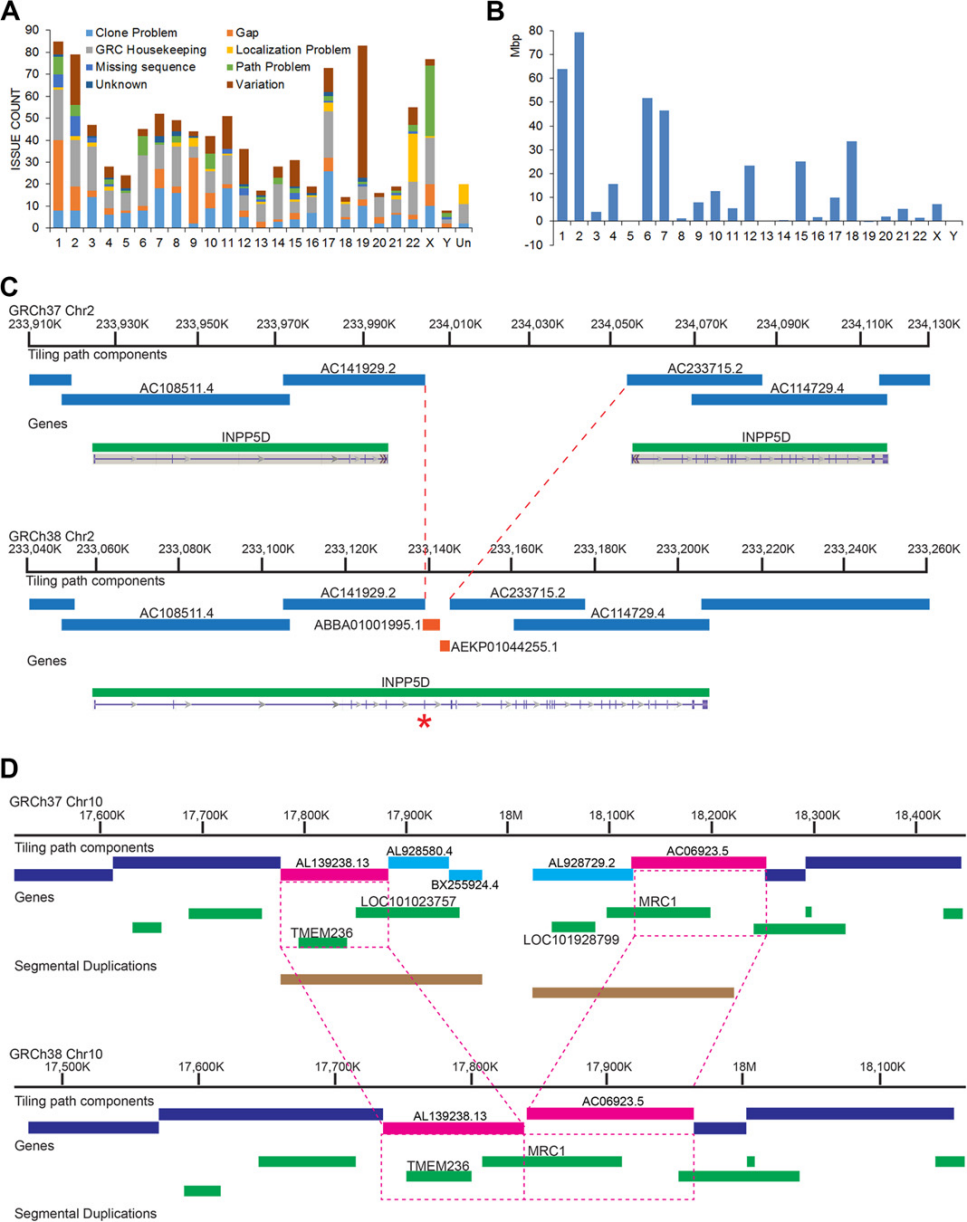
Summary of GRCh38 updates. (*A*) Chart showing issues resolved for GRCh38 on each chromosome by issue type. Each issue represents a unique assembly evaluation and corresponding curation decision. (*B*) Changes in placed scaffold N50 length from GRCh37 to GRCh38. Changes on Chromosomes 5, 13, 19, and Y are <55 kbp each. (*C*) Addition of whole-genome sequencing components (orange bars) resolves a GRCh37 gap, consolidating the split annotation of *INPP5D* and restoring a missing exon (asterisk) in GRCh38. The default 50-kbp gap in GRCh37 greatly overestimates the actual amount of missing sequence (∼6 kbp). (*D*) Schematic of a curated collapse in GRCh38 Chr 10. Clones from two incompatible haplotypes (pink and light blue) were mixed in the GRCh37 tiling path, creating a false gap and segmental duplication involving the single copy genes *TMEM236* and *MRC1* (*top*). In GRCh38 (*bottom*), clones from the blue haplotype have been eliminated (∼200 kbp), closing the gap and providing the correct gene content.

Upon the release of GRCh37.p13 in June 2013, the cumulative set of 204 patch scaffolds covered 3.15% of the chromosome assemblies, included >7 Mb of novel sequence, and met previously defined GRC criteria for the trigger of a major assembly release (Church et al. 2011). We submitted GRCh38, a coordinate changing update of the human reference assembly, to the International Nucleotide Sequence Database Collaboration (INSDC) in December 2013 (GCA_000001405.15). Because the reference remains under active curation, we have subsequently provided quarterly GRCh38 patch releases, which do not affect the chromosome coordinates, the latest of which was GRCh38.p10 (GCA_ 000001405.25). The initial GRCh38 release represents the resolution of more than 1000 issues reported to the GRC tracking system, spanning all chromosomes and encompassing a variety of problem types, including gaps, component and tiling path errors, and variant representation (https://www.ncbi.nlm.nih.gov/projects/genome/assembly/grc/human/issues/) (Fig. 1). Genome-wide alignments of GRCh38 to GRCh37 reveal 11 Mb (0.37% of total length) of inverted sequence, whereas 75 Mb (2.3% of total length) of ungapped sequence in the new assembly has no alignment to GRCh37 (Supplemental Worksheet S3). In contrast, only 5 Mb (0.17%) of ungapped GRCh37 sequence has no alignment to GRCh38. As in previous assembly updates, we used finished, clone-based components for assembly updates wherever possible because of their high per-base accuracy and haploid representation of actual human sequence. With >95% of the chromosome total sequence and 98% of noncentromeric sequence derived from genomic clone components, the GRCh38 reference assembly chromosomes continue to provide a mosaic haploid representation of the human genome, rather than a consensus haploid representation. The sequence contribution from RP11, an anonymous male donor of likely African-European admixed ancestry, remains dominant (∼70%), but has decreased by ∼1.5% relative to the previous assembly version (Supplemental Fig. S1; Green et al. 2010, Supplementary Online Materials 16).

Table 1 summarizes the GRCh38 assembly statistics of length, N50 and gaps relative to GRCh37, and several recently generated de novo assemblies. The GRCh38 assembly is longer and more contiguous than previous reference assembly versions (https://www.ncbi.nlm.nih.gov/projects/genome/assembly/grc/human/data/) (Fig. 1; Table 1). Although the total number of reference assembly gaps grew, increases occur when sequence added into a preexisting gap is not contiguous with either gap edge or when sequence additions are comprised of scaffolded whole-genome sequencing (WGS) contigs. The increase in gap count in GRCh38 is largely attributable to the replacement of the single centromere gap in each chromosome with scaffolds of modeled sequence (described below), and WGS sequences flank more unspanned gaps and spanned gaps in GRCh38 than in GRCh37 (Supplemental Table S1). For more details of assembly gaps, see the Supplemental Notes and Supplemental Table S2.

**Table 1.**
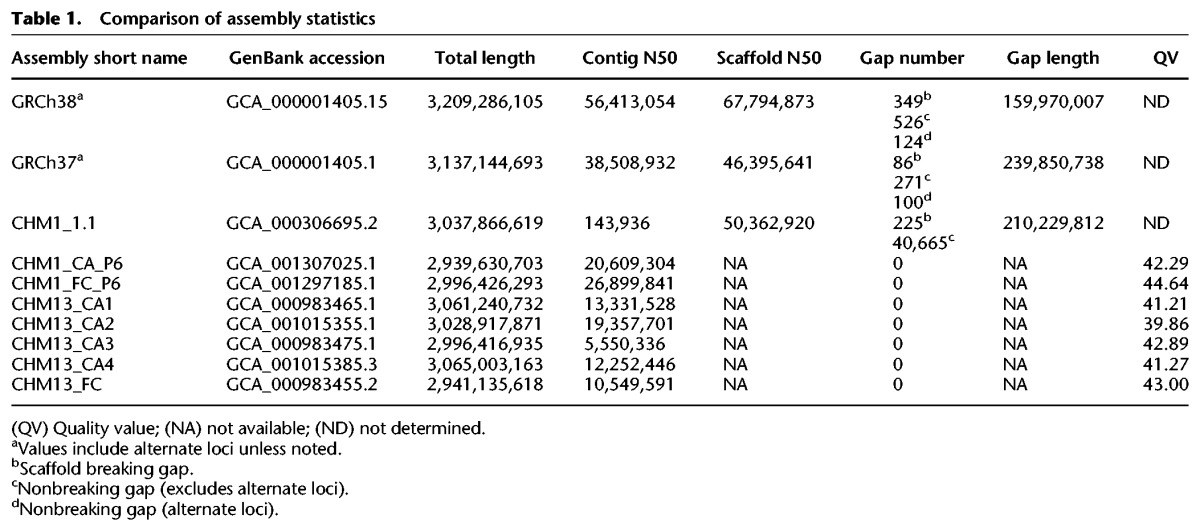
Comparison of assembly statistics

The suite of updates provided in the GRCh38 assembly had a positive impact on assembly annotation. Comparison of the NCBI *Homo sapiens* annotation release 105 of GRCh37.p13 (https://www.ncbi.nlm.nih.gov/genome/annotation_euk/Homo_sapiens/105/) and annotation release 106 of GRCh38 (https://www.ncbi.nlm.nih.gov/genome/annotation_euk/Homo_sapiens/106/) shows an increase in the numbers of genes and protein coding transcripts, with a concomitant decrease in partially represented coding sequences and transcripts split over assembly gaps (Fig. 1; Table 2). Because the transcript content of these two annotation releases was not identical and may contribute to observed differences in the annotation statistics, we also aligned two large public annotation sets (GENCODE23 [basic] and RefSeq71) to the GRCh37 and GRCh38 full assemblies to gauge the impact of improvements on gene representation (Harrow et al. 2012; O'Leary et al. 2016). Similar to the previously described comparison, in GRCh38 we find that both annotation sets show increases in overall transcript alignments with a substantial decrease in split and low quality transcript alignments (Table 3; Supplemental Worksheet S1). We looked at the intersection of the transcripts with problematic alignments with two clinically relevant gene lists: a set of genes enriched for de novo loss of function mutations identified in Autism Spectrum Disorder (*n* = 1003) (Samocha et al. 2014) and a collection of genes preliminarily proposed for the development of a medical exome kit (*n* = 4623) (https://www.genomeweb.com/sequencing/emory-chop-harvard-develop-medical-exome-kit-complete-coverage-5k-disease-associ). Among the set of RefSeq transcripts with problematic alignments to GRCh37, we observed six gene overlaps with the former and 14 with the latter, whereas we found six and 22 for the GENCODE cohort (Supplemental Worksheet S1). The majority of these genes (RefSeq: *n* = 6/6 and *n* = 9/14 and GENCODE: *n* = 5/6 and *n* = 9/22, respectively) are no longer associated with transcript alignment issues in GRCh38, suggesting the newer assembly is a better substrate for clinical studies.

**Table 2.**
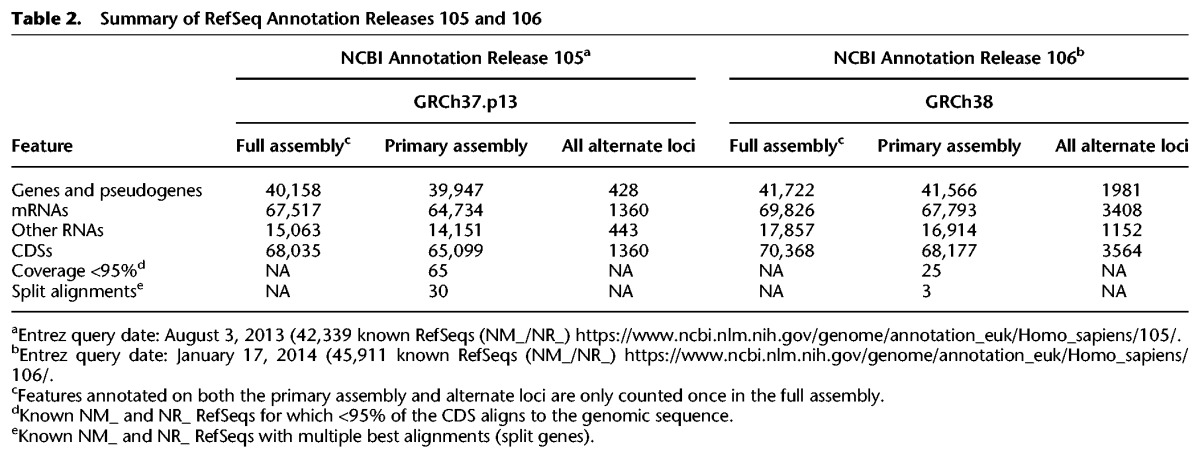
Summary of RefSeq Annotation Releases 105 and 106

**Table 3.**
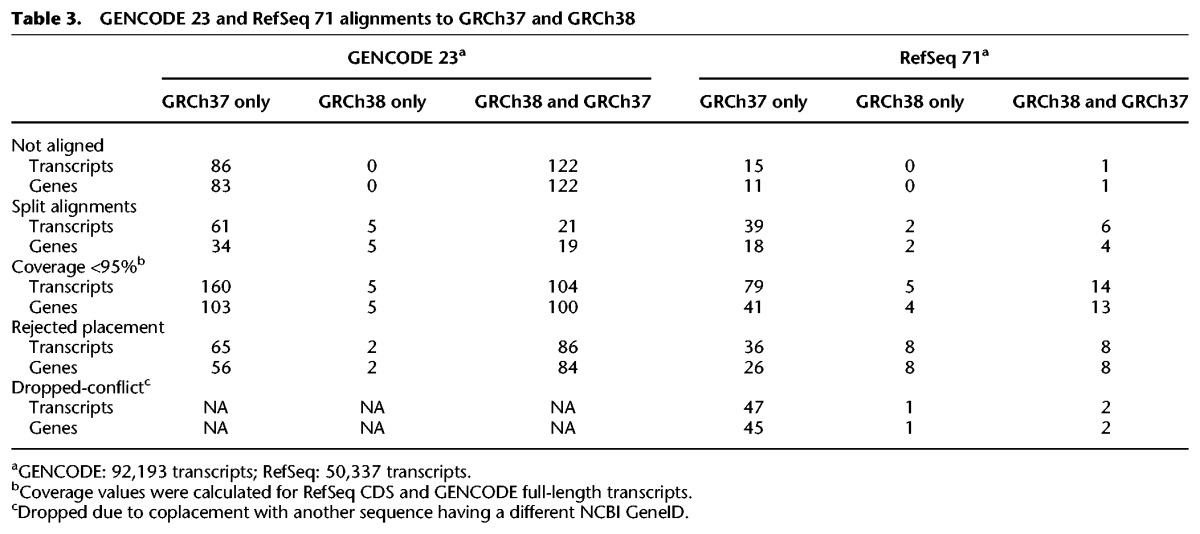
GENCODE 23 and RefSeq 71 alignments to GRCh37 and GRCh38

### Centromeres

A major change in the content of the reference genome assembly is the replacement of the 3-Mbp centromeric gaps on all GRCh37 chromosomes with modeled centromeres from the LinearCen1.1 (normalized) assembly, derived from a database of centromeric sequences from the HuRef genome (GCA_000442335.2) (Supplemental Methods; Levy et al. 2007; Miga et al. 2014). We added the modeled centromeres to the reference assembly to serve as catalysts for analyses of these biologically important and highly variant genomic regions, as annotation targets, and to act as read sinks for centromere-containing reads in mapping analyses (Miga et al. 2015). Consistent with our reasoning that such sequences may improve read alignments, 21.7% (by length) of the “decoy” sequence used in the 1000 Genomes Project to reduce spurious read mapping, and previously shown to improve variant calling (Li 2014), was identified by RepeatMasker as alpha-satellite centromeric repeat (ftp://ftp.1000genomes.ebi.ac.uk/vol1/ftp/technical/reference/phase2_reference_assembly_sequence/) (The 1000 Genomes Project Consortium 2015). Each centromere model represents the variants and monomer ordering of the chromosome-specific alpha-satellite repeats in a manner proportional to that observed in the initial read database, but the long-range ordering of repeats is inferred. In contrast to the remainder of the chromosome sequence, in which each underlying clone component represents the actual haplotype of its source DNA, the modeled sequence is not an actual haplotype, but an averaged representation. The GRCh38 modeled centromeres also contain largely unordered and unoriented islands of euchromatic sequences that are taken from the same collection of HuRef sequences, as well as from genomic clones. One such island, in the modeled centromere for Chromosome 3, provides reference representation for a *PRIM2* paralog (NCBI gene LOC101930420) that was missing in GRCh37 (Genovese et al. 2013a,b). Due to the modeled nature of these sequence representations, we suggest that variant and other analyses within these regions be treated independently of similar analyses made elsewhere in the genome. We anticipate that these modeled sequences will be updated in future assembly versions as new sequencing and assembly technologies make it possible to provide longer-range representations for these regions.

### Retiling

Although a subset of missing sequences is associated with gaps deemed recalcitrant to cloning, segmental duplications or other complex genomic architectures are implicated in most remaining gaps or misassemblies (Bailey et al. 2001; Sharp et al. 2005; Chaisson et al. 2015a). In collaboration with various external groups, we identified and investigated reported path issues and associated assembly gaps using a combination of techniques, including optical maps (Teague et al. 2010; Howe and Wood 2015), Strand-seq (Falconer et al. 2012), admixture mapping (Genovese et al. 2013a) and reevaluation of component sequences and overlaps (Mueller et al. 2013). These analyses uncovered some substantial misassemblies in GRCh37 that spanned several megabases and many genes, including the regions at 1q21, 10q11, and a peri-centromeric inversion of Chromosome 9. Although we were able to improve or resolve some path problems through reordering of existing assembly components to match optical maps, we found that other approaches were needed at more complex regions where allelic and paralogous variation made it impossible to confidently define paths with clones representing a mosaic of diploid DNA sources. In these instances, we replaced GRCh37 components with new tiling paths comprised of BAC clones representing the single haplotype of the essentially haploid CHM1 genome (Dennis et al. 2012; Steinberg et al. 2014), or on Chromosome X, with the single haplotype represented in RP11 (Mueller et al. 2013). We also retiled several genomic loci associated with immune responses (*IGK*, *IGH*, *LRC*-*KIR*, and the cytokine cluster on 17q) with CHM1 clones, replacing the unvalidated mosaic representations in GRCh37 and previous assembly versions to ensure the reference-provided representations of these clinically important regions that actually exist in the human population (Supplemental Worksheet S2; Watson et al. 2013, 2015). Many of these improvements were made public before the release of GRCh38, with 67 of the 131 GRCh37 fix patch scaffolds addressing errors associated with mixed or expanded haplotypes. It is important to note that these new representations may not always be common across any or all populations. Wherever possible, we preserved the assembly representation of genes for which the CHM1 haplotype is deleted by adding components containing these genes to alternate loci scaffolds. Resolution of tiling path issues and assembly gaps is not always accompanied by sequence addition or replacement. For example, we removed three components on Chromosome 10, representing ∼200 kbp of falsely duplicated sequence, to close a gap and correct gene representation (Fig. 1). Ongoing reference assembly curation efforts include providing haplotype resolved paths at other complex loci, such as the Prader-Willi and flanking regions at 15q11-13 (Antonacci et al. 2014).

### Paralogous sequence additions

**Figure 2.**
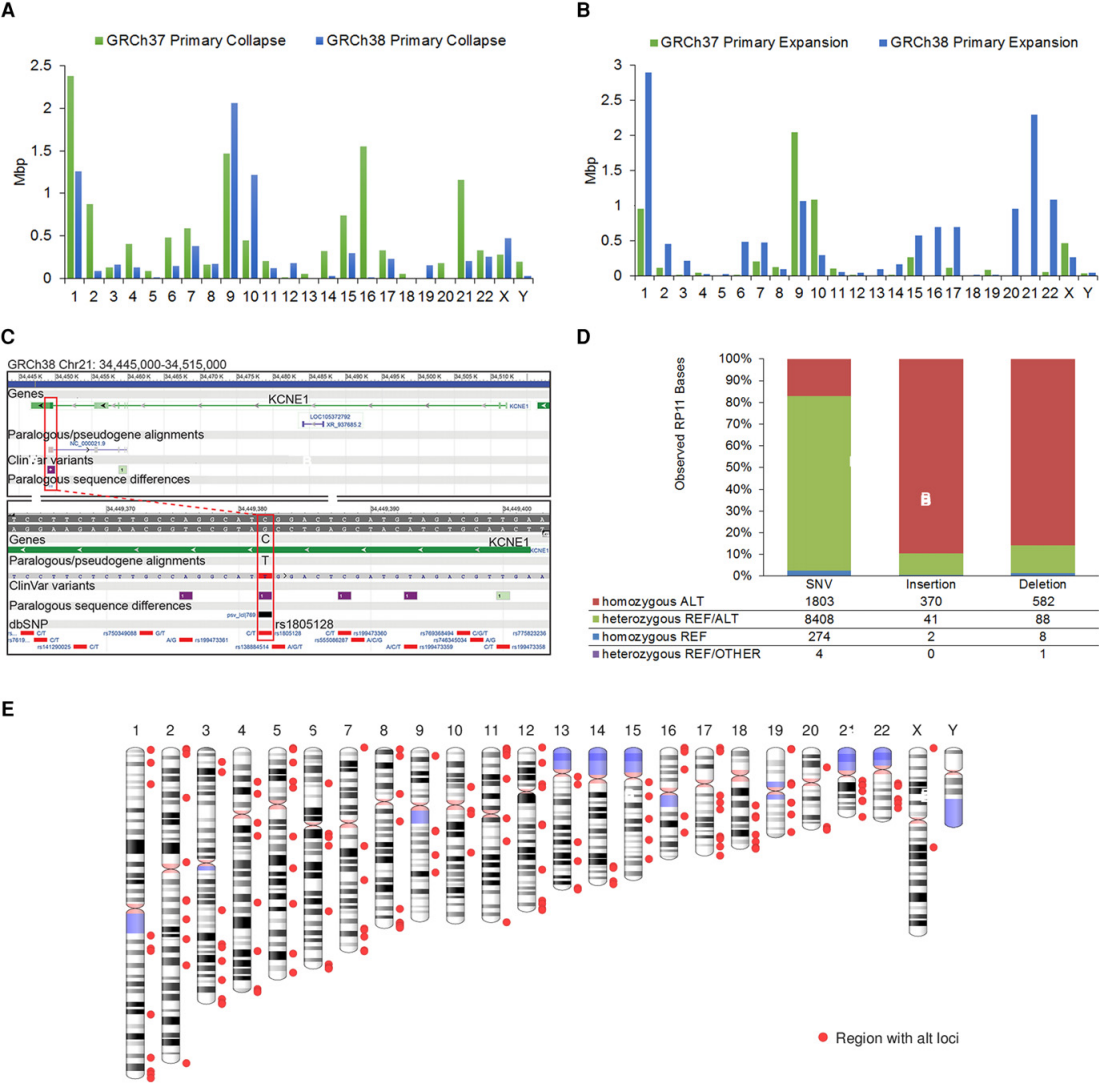
Evaluation of assembly updates. (*A*,*B*) Plots showing the per-chromosome lengths of sequence collapse (*A*) and expansion (*B*) of the GRCh37 (green) and GRCh38 (blue) primary assembly units (from which alternate loci are excluded), based on their assembly–assembly alignment. (*C*) Browser view of *KCNE1* on GRCh38 Chr 21. The *lower* panel shows a zoomed view of the *top*, illustrating a paralogous sequence alignment and paralogous variant (psv) overlapping SNP rs1805128 (red box), a putatively pathogenic ClinVar variant we observed remapping to multiple locations in GRCh38, due to the addition of paralogous sequence. Because previous assembly versions lack this paralog, reads may map incorrectly in this region, and the pathogenicity of the variant and associated diagnostic calls should not be based only on such analyses. (*D*) Plot showing the allele distribution in RP11 WGS reads for the set of GRCh37 bases located in RP11 assembly components that were flagged as putative errors because they were not observed in the 1000 Genomes phase 1 data set. (*E*) Ideogram showing the distribution of regions containing alternate loci scaffolds in GRCh38.

In the course of closing gaps and correcting path errors, we focused on providing reference assembly representation for previously missing human-specific and paralogous sequences. More than 100 segmentally duplicated regions have been estimated to be underrepresented in previous versions of the reference assembly (Sudmant et al. 2010). We have previously shown that an incomplete reference assembly can lead to incorrect mapping of reads (Church et al. 2011), which could subsequently lead to misidentifying paralogous sequence variants as allelic sequence variants. With reported regions as a guide, we used whole-genome maps, admixture mapping, and FISH and alignment analyses to resolve misassemblies and identify and localize components in the assembly. To evaluate our efforts, we analyzed NCBI assembly–assembly alignments of GRCh37 and GRCh38 to determine the relative extents of expansion and collapse in the two assemblies. The NCBI alignment protocol produces outputs that include both reciprocal best hits and nonreciprocal best hits (Steinberg et al. 2014; Kitts et al. 2016). For a given assembly in an alignment pair, genomic regions exhibiting both types of alignments are considered collapsed relative to the other assembly, whereas those with only nonreciprocal best-hit alignments are considered expanded (https://www.ncbi.nlm.nih.gov/genome/tools/remap/docs/alignments). Evaluating the lengths of collapsed and expanded sequence on the chromosomes in both assemblies, we observed that all GRCh37 chromosomes exhibit more collapse than their GRCh38 counterparts (Fig. 2). The increased variant representation in GRCh38 is responsible for much of this, as GRCh38 alternate loci scaffolds are implicated in the alignments of the 10 largest GRCh37 collapsed regions, as well the 10 largest GRCh38 expanded regions (Supplemental Worksheet S3). To assess the relative collapse and expansion of the two assemblies independent of the alternate loci, we compared the alignments of the nonredundant collection of sequences comprising the chromosomes and unlocalized and unplaced scaffolds (primary assembly units). Consistent with the full assembly alignments, we find that nearly all GRCh37 chromosomes exhibit a greater degree of collapse and less expansion than their GRCh38 counterpart; we also observe a correspondence between the most collapsed GRCh37 and most expanded GRCh38 assembly regions (Fig. 2; Supplemental Fig. S2). From these analyses, we find that not only does the GRCh38 assembly gain additional sequence representation through the addition of alternate loci, but the GRCh38 chromosomes provide more accurate representations of duplicated or paralogous regions than those of GRCh37.

To assess the implications of these expanded sequences, we examined their effect at GRCh37 and GRCh38 genomic sites annotated with the subset of dbSNP Build 147 variations described in ClinVar (Landrum et al. 2014). In one analysis, we aligned reads from the Ashkenazi female sample NA24143 (Zook et al. 2016) with BWA-MEM (Li 2013) and evaluated ClinVar sites that have coverage with at least one MAPQ 20 or greater alignment in the GRCh37 and GRCh38 primary assemblies. Of 1525 sites lacking MAPQ 20 coverage in GRCh37, we found variants annotated at 10 locations, representing three different chromosomes, which gained such coverage in GRCh38 (Table 4). Each of these regions was explicitly curated to remove redundant sequence or correct haplotype expansions in GRCh37. Variant calls missed on GRCh37 at these locations due to the artificial presence of confounding sequence should now be possible to call on GRCh38. We also identified variants annotated at 135 locations, associated with six different genomic regions, at which such coverage was lost in GRCh38. All are correlated with GRC curations in which allelic or paralogous sequence was added in GRCh38, suggesting that read alignments at these loci in GRCh37 may give rise to false variant calls. Together, these analyses show that assembly updates associated with the representation of duplicated or paralogous sequence affect read alignment, including at clinically relevant loci, which may have critical impacts on variant discovery and diagnosis.

**Table 4.**
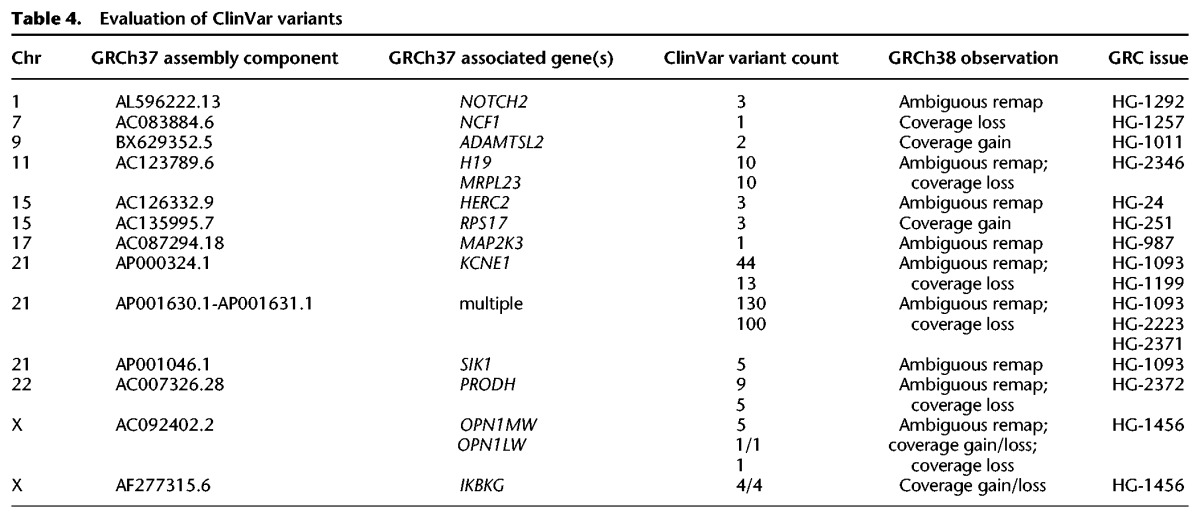
Evaluation of ClinVar variants

In a second analysis, we used the same collection of ClinVar variants (*n* = 113,368) to evaluate the impact of assembly updates on the remapping of data from GRCh37 to GRCh38. We identified a subset of unique GRCh37 ClinVar variants (*n* = 210), including at least one described as putatively pathogenic, which mapped ambiguously to the GRCh38 primary assembly. These variants are associated with nine genomic regions, all of which underwent deliberate curation to add sequence deemed missing from previous assembly versions (Table 4; Supplemental Worksheet S4). In some instances, the newly added sequence exhibits paralogous variation and represents what was previously declared to be the nonreference allele (Fig. 2). The results from this limited survey of human variation further illustrate the potential impact that assembly updates can have on variant calling and diagnosis and demonstrate the importance of performing such evaluations on the GRCh38 assembly, with its expanded sequence representation.

### Base updates

In addition to large-scale curations, we also performed targeted sequence updates. Because erroneous reference bases, estimated to occur at a rate of 10^−5^ (International Human Genome Sequencing Consortium 2004), can result in incorrect variant calls, complicate gene annotation, and in the case of indels, complicate read alignments, we sought to identify and correct such sites (International Human Genome Sequencing Consortium 2004). We considered a set of 15,244 GRCh37 single-nucleotide variants (SNVs) and 2375 indels with a minor allele frequency (MAF) = 0 in the phase 1 analysis of the 1000 Genomes Project or that were identified in a *k*-mer analysis as candidate reference errors (Supplemental Methods; The 1000 Genomes Project Consortium 2010, 2012). For the subset of sites located in RP11 BAC components (*n* = 11,581), we sought to validate the assertion that the reference alleles represent errors. We examined allele distributions in the RP11 genome by aligning Illumina WGS reads from RP11 (SRR834589) and looking for evidence of the reference base in the sample. Among the candidate sites, we observed that 80% of SNVs, 10% of insertions, and 13% of deletions were heterozygous in RP11 (Fig. 2), indicating that they were not reference errors. This analysis demonstrates the difficulty in distinguishing private or very low frequency alleles from error, even with large variation data sets. To ensure we retained the haplotype structure of the RP11 BAC components in the reference assembly, we did not update the observed RP11-derived heterozygous candidate sites in GRCh38. Given the admixed ancestry of the RP11 donor, it remains to be determined whether these otherwise unknown alleles are preferentially associated with a specific population background. If they are on the African haplotype, their elimination might inadvertently remove variants found in populations not represented in the 1000 Genomes Project.

For the remaining sites, we used reads from samples in the 1000 Genomes phase 1 data set or RP11 to generate short WGS contigs whose sequence overlapped the target site and surrounding bases (Supplemental Methods). We validated these “mini-contigs” by alignment to GRCh37, confirming that they differed only at the target site and contained the expected alternate allele, and added them to the assembly. In a small number of cases, WGS contigs from other human assemblies or genomic PCR products were instead used to update bases. We updated an additional 376 sites identified during the course of other curation activity that although not monomorphic, were either deemed universally rare according to 1000 Genomes phase 1 analysis or that had been reported by clinical testing laboratories and annotators to have a substantial negative impact on clinical variation analyses or annotation. In total, 8248 sites were updated (Supplemental VCF S1, VCF S2), 35 of which are annotated as ClinVar variants in GRCh37. These targeted updates represent the first large-scale effort to correct base-pair–level errors in the reference.

### Alternate loci additions

In addition to adding sequence at assembly gaps and providing representation for missing copies of segmental duplication, we increased the number of alternate loci scaffolds to provide more representation for population variation in the reference. GRCh38 includes 261 scaffolds representing 178 genomic regions (Fig. 2). As described previously, these alternate loci improve read mapping, provide the only reference representation for more than 150 genes, and capture sequence from the 1000 Genomes “decoy” used as a read sink for GRCh37, endowing it with chromosome context (Church et al. 2011, 2015; The 1000 Genomes Project Consortium 2015). Of particular note, GRCh38 includes 35 different representations for the immune-related leukocyte receptor complex on Chromosome 19 (Pyo et al. 2010) and two additional haplotype resolved paths of the highly variable and complex *SMN1-*containing spinal muscular atrophy (SMA) region on Chromosome 5 (Schmutz et al. 2004). The GRC website provides additional information about alternate loci with a series of region-specific pages that provide a graphical display and a report of associated curation issues (https://www.ncbi.nlm.nih.gov/projects/genome/assembly/grc/human/).

### Impacts on read mapping

**Figure 3.**
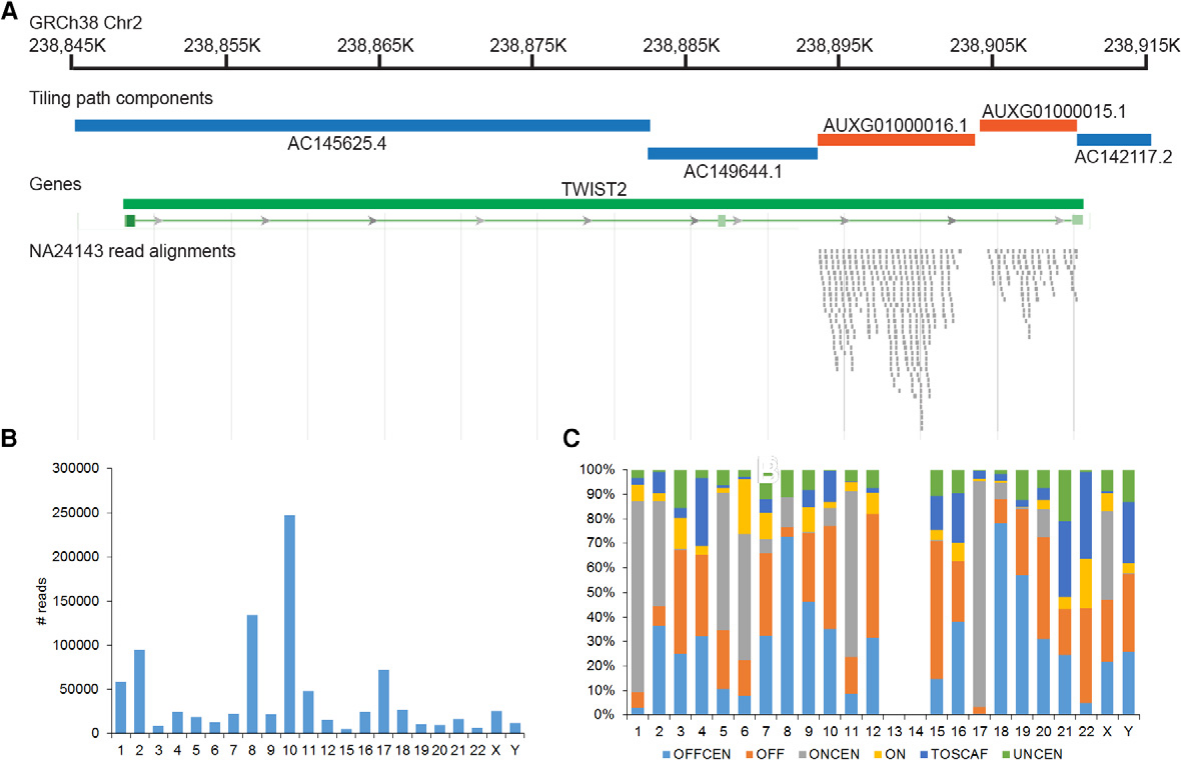
NA24143 read alignments to GRCh38. (*A*) Schematic showing the alignment of a subset of reads unmapped on GRCh37 to GRCh38. Reads align to GRCh38 at the position of components that were added to span a GRCh37 assembly gap (orange). (*B*) Graph showing counts of reads uniquely mapped to unchanged regions of GRCh37 that uniquely map to nonequivalent locations in GRCh38. (*C*) Chart describing the GRCh38 distribution of reads from *B*, categorized by sequence location (same or different chromosome/scaffold) and sequence type (centromeric versus noncentromeric): (OFFCEN) movement to centromeric sequence on a different chromosome; (OFF) movement to noncentromeric sequence on a different chromosome; (ONCEN) movement to centromeric sequence on the same chromosome; (ON) movement to noncentromeric sequence on the same chromosome; (TOSCAF) movement to a noncentromeric unlocalized or unplaced scaffold; (UNCEN) movement to an unplaced scaffold containing centromere-associated sequence.

We evaluated the impact of the cumulative set of GRCh38 updates on read mapping. Reads from the Ashkenazi sample NA24143 used for the ClinVar analysis were aligned to the GRCh37 and GRCh38 primary assemblies and to the GRCh38 full assembly (Supplemental Methods). Although the GRCh37 primary assembly is an excellent mapping target, with 99.92% of reads aligned, we find that 64.32% of the unmapped reads are now mapped to the GRCh38 primary assembly. Consistent with the assembly curation effort, we observe many of these previously unmapped reads aligning to new sequences added at GRCh37 gaps (Fig. 3). This demonstrates that the updates found on the GRCh38 reference assembly chromosomes make them a more robust substrate for analyses than the previous assembly version. We also find that 23.71% of reads that are still unmapped on the GRCh38 primary assembly map to the GRCh38 full assembly, which includes the alternate loci. We frequently observe these reads aligning to sequence unique to the alternate loci, validating GRC efforts to expand reference sequence representation with alternate loci (Supplemental Fig. S3).

Although assembly updates are expected to alter read alignments in changed regions, we also investigated their impact on read mappings in the 2.6 Gbp of unchanged reference sequence, using a script written for this purpose (Supplemental Code). We find that 4.19% of read pairs that map uniquely, albeit imperfectly, to the GRCh37 primary assembly in an unchanged assembly region move to a new location with a different underlying assembly component in GRCh38. Approximately one-third of these moved pairs are also uniquely mapped to GRCh38 (Supplemental Table S3). We also analyzed the movement of individual reads from the moved pairs with respect to location (on- or off-chromosome) and sequence type (centromeric or noncentromeric). We find that both the extent and patterns of read movement are unique to each chromosome (Fig. 3; Supplemental Fig. S3; Supplemental Tables S4, S5). Consistent with a nonrandom pattern of movement, we observe distinct pairings of assembly components overrepresented as GRCh37 and GRCh38 mapping targets for each chromosome. Among reads belonging to moved pairs that also map uniquely to GRCh38, transitions to the modeled and unplaced GRCh38 centromere sequences predominate, but shifts to noncentromeric sequence still account for ∼25% of total movement (Fig. 3; Supplemental Table S4). Together, these analyses demonstrate that the assembly updates and alternate loci in GRCh38 not only make it a more complete mapping target, but that updates also exert an effect beyond their borders. As a result, we recommend use of GRCh38 for new genome-wide analyses in addition to studies specifically associated with changed regions.

### De novo assembly evaluations

The majority of reference assembly updates in GRCh38 used finished genomic clones. New reference-quality sequence sources are needed, because generation of finished sequence from clone libraries is in significant decline due to cost and some remaining assembly gaps occur in regions recalcitrant to cloning. A growing collection of human genomes in INSDC databases, a prerequisite for any sequence that will contribute to the reference assembly, that were sequenced and assembled with new technologies are candidates for use in assembly improvement (Earl et al. 2011; Vezzi et al. 2012; Bradnam et al. 2013; English et al. 2015; Pendleton et al. 2015; Seo et al. 2016; Shi et al. 2016; Zook et al. 2016). However, WGS assembly sequences have historically not been considered reference quality, raising concerns about their use in reference genome assembly curation. The essentially homozygous genomes of CHM1 and CHM13 have great potential for use in future updates due to the proven usefulness of haploid resources in resolving complex regions (Huddleston et al. 2014; Steinberg et al. 2014; Berlin et al. 2015; Chaisson et al. 2015a). We therefore generated the first collection of WGS de novo assemblies of CHM1 and CHM13 from two new sets of publicly available read data (SRP044331 and SRP051383), using both FALCON (Chin et al. 2016) and Celera Assembler (Berlin et al. 2015), with the intention of evaluating them with respect to reference assembly characteristics (Supplemental Methods; Supplemental Figs. S4, S5; Supplemental Table S6). We initially compared basic statistics for these assemblies to each other and to the GRCh38 assembly (Table 1; Supplemental Table S7). In addition, we compared the CHM1 assemblies to CHM1_1.1, a hybrid clone and short-read-based reference guided assembly of CHM1 (Steinberg et al. 2014). We used Illumina data to determine the QV scores for each de novo assembly, providing a measure of base-pair–level accuracy. All assemblies exhibit overall high quality, each with a QV near or above 40. For both samples, we found that total lengths of the new assemblies were consistent with respect to one another and to GRCh38 or CHM1_1.1. The contig N50s of the new assemblies exhibited more variability, demonstrating that although all assemblies will have most of the same sequence for a given sample, they vary in how it is put together. Strikingly, even without scaffolding, many of these N50s are comparable with the scaffolded N50s of other recently published de novo WGS assemblies, whereas scaffolding with optical map data led to their near doubling (Supplemental Notes; Supplemental Table S8). In conjunction with additional optical map analyses and BAC paired-end alignments (Supplemental Notes; Supplemental Tables S9, S10) demonstrating long-range assembly accuracy, these data augur well for their ability to contribute to gap closure curation efforts (Wang et al. 2008; Berlin et al. 2015; Chaisson et al. 2015a; Pendleton et al. 2015).

**Figure 4.**
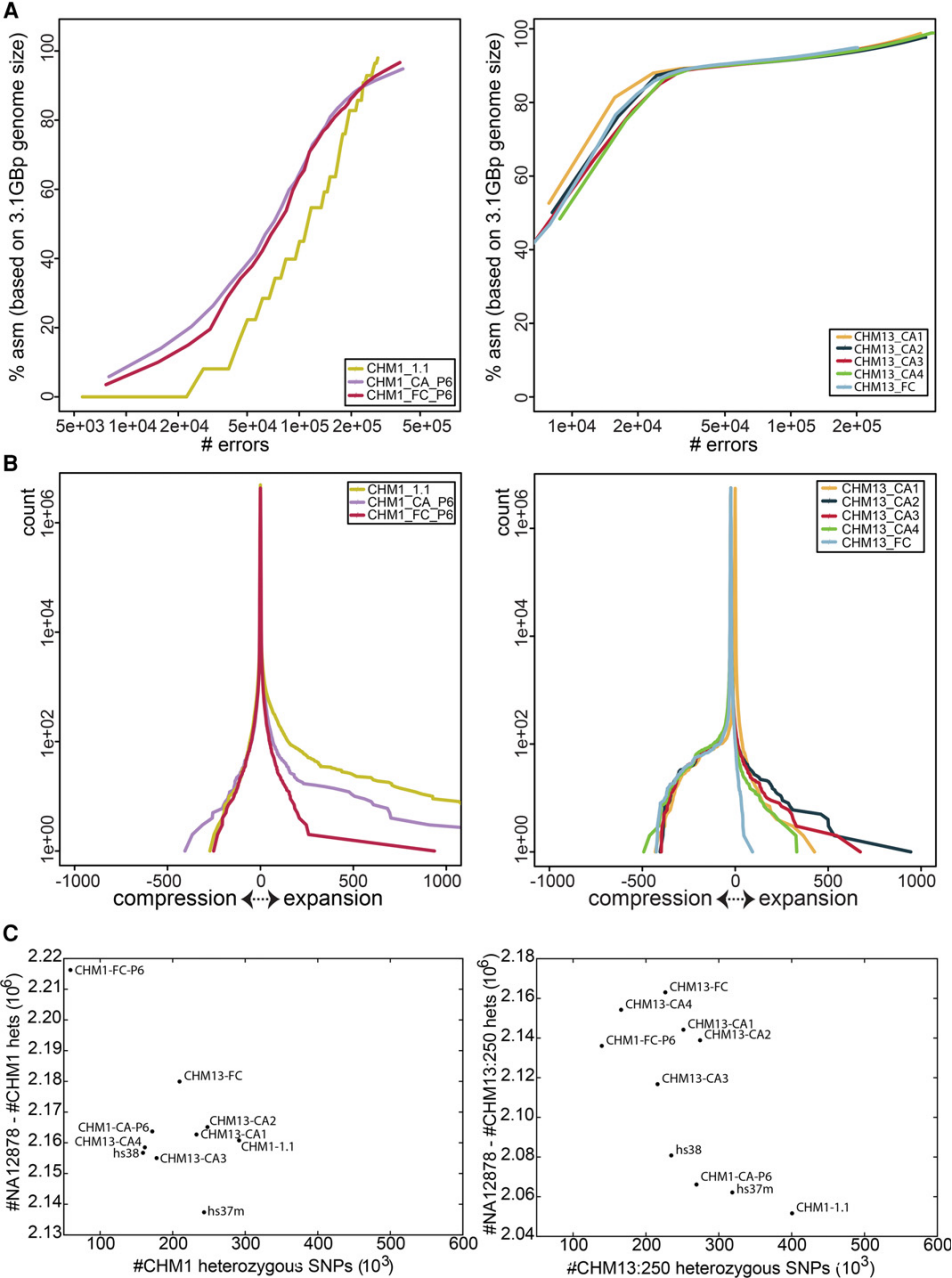
Evaluation of CHM1 and CHM13 assemblies. (*A*) FRC error curve for CHM1 (*left*) and CHM13 (*right*) assemblies. CHM1_1.1 is provided for comparison with the CHM1 de novo assemblies. The *x*-axis is log-scaled. (*B*) FRC compression-expansion curve for CHM1 (*left*) and CHM13 (*right*) showing the distribution of mapped reads. Divergence from the center indicates compression (negative) and expansion (positive). (*C*) Heterozygous SNPs called on the CHM1 and CHM13 de novo assemblies, CHM1_1.1 and GRCh38 using NA12878 and CHM1 (*left*) and CHM13 (*right*) aligned FermiKit assemblies. The *x*-axis represents potential false positives, and the *y*-axis measures potential true positives; optimal assemblies appear in the *upper left* of the plot.

We further evaluated assembly quality with feature response curves (FRC) generated with mapped Illumina read pairs as input to FRC^bam^ (Fig. 4; Supplemental Table S11; Vezzi et al. 2012). Although N50s differ by more than a factor of two among the assemblies, all FRC scores are high and comparable, indicating their overall quality, and additional joins in assemblies with longer N50s do not introduce significant error. However, because repetitive sequences have typically been prone to collapse in WGS assemblies, we also used FRC curves to evaluate compression and expansion in each of the assemblies. Once again, we see that all assemblies fared well with respect to this metric, clustered at the center, with only minor differences between assemblers or parameters for a given sample. The long reads and lack of allelic variation in these new assemblies likely underlie these observations (Huddleston et al. 2014).

We also appraised the assemblies by variant calling with FermiKit, in which heterozygous variant calls based on alignment of haploid samples are considered false positives, likely caused by assembly collapse of tandem repeats and/or segmental duplications (Fig. 4; Supplemental Material; Supplemental Fig. S6; Li 2014, 2015). Heterozygous calls on the collections of CHM1 and CHM13 assemblies were measured using three different haploid de novo assemblies and evaluated with respect to heterozygous calls from the diploid NA12878 sample. These analyses uniformly show that for the CHM1 sample, the FALCON-based assembly is a better substrate for variant calling, but also suggest that Celera Assembler produces a better variant calling substrate for the CHM13 sample. Comparison to GRCh37 and GRCh38 suggests that these new haploid assemblies may serve as more reliable substrates for variant calling than the reference assembly, although further analysis is needed to determine whether improvements occur in genomic regions of interest. However, because variant calling is only one use case for the reference assembly, we also examined other facets of these de novo assemblies.

Gene content is another important metric for assembly quality, especially if the assembly will be used as an annotation substrate. We examined three aspects of RefSeq transcript alignments to the CHM1 and CHM13 assemblies to assess different aspects of assembly quality. Total gene representation reflects overall assembly quality and content, coplacement of genes reflects collapsed segmental duplications, and frameshift analysis provides information about the accuracy of gene representation within the assembly (Table 5). We find that all assemblies compare favorably to each other and to GRCh38 with respect to total content of gene representation. In contrast, we find that all CHM1 and CHM13 assemblies exhibit a substantially greater number of transcripts that are dropped due to conflicting placement with transcripts representing other genes, compared both to the GRCh38 reference assembly and to the CHM1_1.1 assembly (Table 5). The genes associated with coplaced transcripts are largely shared within and between assemblies derived from CHM1 or CHM13 and are dominated by paralogous genes, many of which reside in multimegabase, highly complex, and/or segmentally duplicated regions (Supplemental Worksheets S5, S6; Supplemental_ GFF3_S1.tar.gz; Supplemental_GFF3_S2.tar.gz). The genomic locations associated with the transcripts on these lists may reflect regions still recalcitrant to assembly with current read lengths and algorithms. These lists also include haplotype-specific or copy-number variant genes, for which coplacement occurs when they are absent from the sample haplotype. In contrast to the GRCh38 reference assembly, in which alternate loci provide representation for multiple haplotypes at many loci, the CHM1 and CHM13 samples represent only a single haplotype and are expected to have a slightly lower overall gene content, which may also contribute to the higher number of coplaced genes on these assemblies relative to GRCh38. However, there are 35%–40% fewer transcripts dropped from the CHM1_1.1 assembly due to coplacement than from the FALCON or Celera Assembler CHM1 assemblies, indicating that assembly method has a substantial impact on gene representation. In the context of reference assemblies, these findings demonstrate that caution is required when using assemblies that have been deemed “high quality.” Gene content must be considered as part of the determination of whether an assembly is suitable for use as a reference or in reference curation.

**Table 5.**
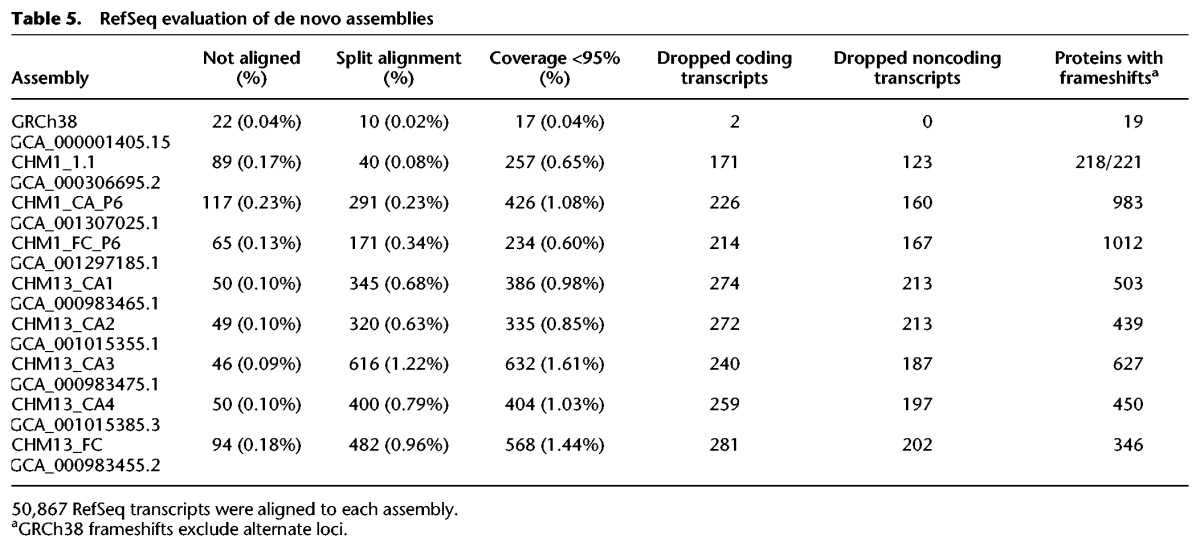
RefSeq evaluation of de novo assemblies

Assembly method can have a striking impact on the accuracy of predicted proteins, as can sequencing technology (Florea et al. 2011). To assess the quality of protein representation in these assemblies, we identified RefSeq alignments containing frameshifting (FS) indels in coding sequence. We observe that the number of transcripts aligning with frameshifting indels is much higher in these new assemblies compared to GRCh38 or CHM1_1.1 (Table 5). Additionally, for both samples, we find that the likelihood of a FS protein being unique to a particular assembly or shared among all assemblies is roughly equivalent, further confirming the influence of assembly method on protein prediction. Using the subset of FS proteins not common to all assemblies as a denominator, we examined the percentage of uniquely FS proteins in each assembly. For the CHM13 sample, an average of 50% of FS proteins were unique to each assembly, ranging from a high of 61% in the FALCON assembly to a low of 40% in the Celera Assembler assemblies. For CHM1, both assembly methods performed similarly, with ∼50% of FS proteins unique to either assembly. We also looked at the subset of FS proteins common to all de novo assemblies for each sample, which are most likely to represent true variation and/or arise from issues with the read data or genomic regions problematic for all assembly methods. Consistent with the former, we find that the *GRIN3B* gene has a frameshifting indel in all CHM1 and CHM13 assemblies that corresponds to rs10666583, a known inactivating variant associated with susceptibility to schizophrenia (Matsuno et al. 2015). Although further analyses are required to understand the differences at the assembly sequence level and to assess the effect that assembly polishing tools such as Pilon might have (Walker et al. 2014), these data clearly demonstrate the variability in gene representation that can arise due to assembly method. Together, our analyses indicate that recent long read assemblies have good continuity, a low error rate, and a high rate of gene completeness compared to previous de novo efforts. They should prove valuable for resolving a subset of remaining reference assembly gaps and providing variant sequence representations. However, the reference still provides better representations of long repeat structures and genes. Not only do our data demonstrate a continued role and relevance for the current human genome reference assembly, they emphasize the need for continued development in the fields of sequencing and assembly if WGS assemblies are truly to be recognized as reference quality genomes and to ensure the human reference genome of the future exhibits the necessary all-around quality essential to fulfill its many roles in an ever-expanding set of analyses.

## Discussion

The human reference genome assembly, initially released more than a decade ago, remains at the nexus of basic and clinical research. Like the continually changing landscape in which it exists, the reference assembly also evolves. As we have described, GRCh38, the current version of this resource, exhibits improved assembly statistics, contains corrected representations of several large-scale clinically relevant regions, and provides new sequence content. This content both captures previously missing genomic sequence and provides representations of population genomic diversity. The updates to the assembly render it an improved annotation substrate and alter its characteristics as a mapping target. Together, the suite of changes introduced in GRCh38 make it the most complete and accurate representation of the human genome yet produced and we recommend its use over previous assembly versions for all types of analyses.

In order to establish the relevancy of a clone-based reference assembly in the context of new sequencing and assembly technologies, we also generated and evaluated several de novo long read-based assemblies representing the CHM1 and CHM13 haploid genomes with respect to each other and GRCh38. All proved to be high quality and demonstrate the capabilities of FALCON and Celera Assembler to generate robust assemblies from large scale, complex genomic data sets. Nonetheless, each assembly method imparted distinct characteristics to the haploid assemblies, and none could be considered the best genome representation by all metrics evaluated. We suggest that de novo assemblies may be further improved by development to support the use of additional data sets, such as Illumina reads or genomic clones, as input to the assembly process, or by post-processing with various error correcting tools. Technological improvements leading to further increases in read length and scaffolding, or use of longer library inserts should also improve assembly contiguity, particularly in repetitive and/or segmentally duplicated regions, especially when coupled with the complementary use of mapping techniques. Preliminary analyses breaking the GRCh38 assembly at locations with segmental duplications >50 kb reduce the contig N50 from 56 Mb to ∼30 Mb (CS Chin and A Wenger, pers. comm.), illustrating the need for long-range inputs to the assembly process that can span such loci. The de novo assemblies also demonstrate the challenges and limitations in transforming data associated with repetitive or complex genomic regions from a rich graph-based assembler representation to a narrower linear assembly representation. It may be desirable to adjust parameters to convey different aspects of the data, such as length, variation content, or sequence quality, in order to produce assemblies best suited to different types of analyses. Notably, such suites of sequence representations could be captured in the current reference assembly model as alternate loci scaffolds, and de novo assemblies may further contribute to the reference in this way.

Our analysis of GRCh38 and the de novo assemblies demonstrates that the reference assembly remains the most comprehensive and highest quality representation of the human genome, capable of supporting the widest range of analyses and discoveries. However, we also foresee an evolving role for the reference genome assembly in the context of two anticipated sea-changes in genome biology that will be realized by ongoing development for technological and computational methods: (1) a proliferation of reference-quality individual diploid genome assemblies; and (2) a comprehensive graph-based representation of genome-wide population variation. In both contexts, the reference assembly is likely to serve as a point of integration. In an era of personalized medicine, we anticipate the integration of data analyses performed on individual genomes through the reference assembly. Regardless of its quality, an assembly representing an individual genome will be limited in its representation of variation. The reference assembly provides context for both the scale and types of variation that will be observed from one sample to the next. Using the reference in this role presents a mechanism for transferring individual interpretations to populations. However, these efforts will require tools and resources for comparative analysis. Without continued development in this area, the challenges incurred today in evaluating analyses performed on different versions of the reference assembly, or transitioning data sets between them, will persist and be magnified as the extent of the differences between individuals will be considerably greater than those between reference assembly versions. GRCh38, with its robust genome representation and well-characterized assembly features provides the framework for this development.

The reference is also a framework for the establishment of a genome graph that represents population variation. This is a natural step in the evolution of the scientific role of the reference genome assembly. Conceived from the outset as a model of the human pan-genome, the current reference now contains not only chromosome sequences depicting a mosaic of haplotypes from different individuals, but includes alternate loci scaffolds that provide multiallelic and multihaplotypic representation for regions across the genome. Because the alignments that define the relationship of these scaffolds to the chromosomes are integral pieces of the assembly model, we submit that the reference has already started the transition into a graph-based depiction of the human genome. As genome graphs progress further into nonlinear forms, the reference chromosome sequences are well-suited to serve as a central path against which variation is described or annotations are made, whereas the alternate loci provide a subset of high-quality and curated branches (Paten et al. 2014; Dilthey et al. 2015; Nguyen et al. 2015; Novak et al. 2016). The Global Alliance for Genomic Health (GA4GH) are using the GRCh38 assembly with alternate loci in a pilot graph-building project (https://github.com/ga4gh/schemas/wiki/Human-Genome-Variation-Map-[HGVM]-Pilot-Project). Ongoing reference curation efforts are aimed at providing additional representations for genomic diversity and have added more than 45 novel patches since the initial release of GRCh38. The continued improvement of the reference assembly does therefore not put it in conflict with these new models, but instead will serve to improve them as it provides a more robust representation of the sequences and relationships that they will portray.

In an idealized view, the reference assembly should be improved until this critical resource is sufficiently complete that it (1) provides chromosome context for any identified human sequence of 500 bp or greater (Church et al. 2011); (2) enables unambiguous data interpretation at all clinically relevant loci; and (3) introduces no systematic error or bias in genome-wide analyses. The substantial improvements and changes represented in GRCh38 move us closer to this ideal on all three points. The analyses of the high quality de novo haploid CHM1 and CHM13 assemblies show that there may soon be new resources that will bring us even nearer to this goal, and repurposing such high-quality WGS de novo assembly sequence for use in the reference assembly drives down curation costs. However, the challenges in migrating data sets and paucity of tools for working with allelic sequence representations (such as alternate loci and patch scaffolds) presents a barrier to the adoption of new assemblies, despite their improvement over previous versions (Church et al. 2015). Likewise, documentation of the improvements found in GRCh38 (such as offered by this publication) is necessary to promote transition to the latest assembly. Although rough calculations suggest the growth in BAM submissions on GRCh38 to the public NCBI Sequence Read Archive (SRA) between 2015 and 2016 was more than 150× the growth rate of submission on GRCh37, the total number of 2016 public BAM submissions on GRCh38 was only ∼30% of that on GRCh37 (C O'Sullivan, pers. comm.). GRCh38 submissions to dbGaP are also growing, albeit more slowly, consistent with anecdotal reports that many clinical groups have not yet transitioned to the updated assembly. In the European Nucleotide Archive (ENA), a preliminary investigation suggests GRCh38 accounts for 39% of all bulk CRAM (Fritz et al. 2011) submissions from October 2013 through December 2016, whereas GRCh37 accounts for 60% (R Leinonen, pers. comm.). Our ability to address the aforementioned challenges will, in part, define the point at which the reference representation is deemed sufficient on all three goals to render further improvements unwarranted. As the community of reference assembly users draws ever closer to that point, we caution that we must let the biology, rather than the technology or an abstracted goal, be the primary driver for that decision. In keeping with that view, we foresee a continued need for assembly evaluation in the context of the ever-evolving landscape of genome research.

## Methods

### Transcript evaluation of assemblies

Alignments were performed and analyzed as described in the Supplementary Methods of Shi et al. (2016). However, in contrast to the RefSeq transcripts, we evaluated coverage for the GENCODE data over the full transcript, rather than the CDS, because we did not have the CDS information.

### Assembly–assembly alignments

Assemblies were aligned using software version 1.7 of the NCBI pipeline as described in the methods of Steinberg et al. (2014). The alignments and alignment reports are available from the NCBI Remap FTP site (ftp://ftp.ncbi.nlm.nih.gov/pub/remap/Homo_sapiens/1.7/) (Kitts et al. 2016). We evaluated chromosome-level collapse and expansion in these alignments and summarized the reported alignment differences with custom code available in the Supplemental Material (Supplemental Code) and at https://github.com/deannachurch/assembly_alignment/. In these analyses, ungapped assembly regions were defined as those comprised of >50% non-N bases.

### ClinVar variant coverage analysis

We assessed coverage using the GATK DepthOfCoverage tool (McKenna et al. 2010), with the parameter --minMappingQuality 20.

We used the following VCF files containing ClinVar variants on the GRCh37 and GRCh38 assemblies to define the sites at which to assess coverage: ftp://ftp.ncbi.nlm.nih.gov/pub/clinvar/vcf_GRCh37/clinvar_20160502.vcf.gz and ftp://ftp.ncbi.nlm.nih.gov/pub/clinvar/vcf_GRCh38/clinvar_20160502.vcf.gz.

We measured the coverage for Illumina reads from sample NA24143 aligned to the GRCh37 and GRCh38 primary assembly units (described below) at these sites. Sites with zero coverage in GRCh37 were remapped to GRCh38 using the NCBI remapping service with default parameters (https://www.ncbi.nlm.nih.gov/genome/tools/remap/docs/api) (Kitts et al. 2016) and coverage reevaluated. Sites with zero coverage in GRCh38 were remapped to GRCh37, and those with coverage were evaluated.

### ClinVar remapping analysis

We used the NCBI remapping service, with default parameters to remap the following variants from GRCh37 (GCF_000001 405.13) to GRCh38 (GCF_000001405.26): ftp://ftp.ncbi.nlm.nih.gov/pub/clinvar/vcf_GRCh37/archive/2016/clinvar_20160502.vcf.gz. We manually reviewed the subset of variants with multiple remappings in the primary assembly unit.

### Base updates

#### Evaluation of candidate bases in RP11 assembly components

We validated candidate erroneous bases in RP11 components with a pileup analysis of the alignments of RP11 Illumina reads to GRCh37 in SRA run SRR834589. The pileup version was sra-pileup.2.3.2.11 (http://ncbi.github.io/sra-tools/), with the parameter --minmapq 20.

We used a cutoff of 90% to define homozygous and heterozygous reference and alternate allele calls at SNVs and a cutoff of 70% for indels. For indels, all nonhomozygous alternate allele calls were manually reviewed. For SNVs, we manually reviewed all sites in which more than two alleles were called or in which alleles not expected for the corresponding dbSNP variant were reported.

#### WGS mini-contig generation

Software used for mini-contig generation was cortex_con_beta_0.04c (http://cortexassembler.sourceforge.net/). For additional details, see Supplemental Methods.

### Alignment of Illumina reads

Of note, 2 × 150-bp paired reads from Ashkenazim trio sample NA24143 were generated as described in Zook et al. (2016) and were aligned with BWA-MEM to the GRCh37 and GRCh38 assemblies. For additional details, see Supplemental Methods.

### CHM1/CHM13 assembly generation

Assemblies were either generated with Celera Assembler 8.3rc2 (Berlin et al. 2015) or with FALCON, an assembler based on HGAP (Chin et al. 2013, 2016). The read data for the WGS assemblies was previously deposited in the SRA with the following accessions: SRP044331 and SRP051383. For additional assembly details, see Supplemental Methods.

### Clone placements

CH17 clone placements were performed and evaluated as described in Schneider et al. (2013) and Steinberg et al. (2014). On the GCA_001307025.1 assembly, the average insert length was 208,547 and the standard deviation was 19,641. On the GCA_001297185.1 assembly, the average insert length was 208,596 and the standard deviation was 19,718.

### BioNano optical maps

Long CHM1 molecules were nicked and labeled according to the BioNano Genomics IrysPrep protocol and loaded on the IrysChip for genome mapping on the BioNano Genomics Irys System imaging instrument. Image detection, assembly, and genome map alignment were performed using BioNano Genomics IrysSolve software tools. Each of the PacBio sequence assemblies were nicked in silico with BspQI to produce a cmap file, which reports the start and end coordinates and the placement of labels for each contig. BioNano Genomics software tools were then used to align each of the sequence assemblies to the CHM1 or CHM13 genome map, and structural variant (SV) detection software was run to generate the SV and hybrid stats provided in this paper (Supplemental Material).

### De novo assembly evaluation with Illumina read data

SRA accessions for reads used as input to Illumina read-based analyses (QV, FRC^bam^, FermiKit) were the following:

- CHM1: SRR2842672 (FRC), SRR642636-SRR642641 (FermiKit);
- CHM13-125: SRR2088062 and SRR2088063;
- CHM13-250: SRR1997411;
- NA12878: ERR194147 (FermiKit).

For additional details of these analyses, see Supplemental Methods.

## Data access

All assemblies have been deposited in GenBank with the following accession numbers: GRCh38: GCA_000001405.15; WGS assemblies: GCA_001307025.1, GCA_001297185.1, GCA_000983465.1, GCA_001015355.1, GCA_000983475.1, GCA_001015385.3, and GCA_000983455.2. These can be retrieved from the NCBI Assembly database (https://www.ncbi.nlm.nih.gov/assembly/).

## Competing interest statement

Richard Durbin is a member of the Scientific Advisory Board of Dovetail Genomics. Deanna Church is an employee of 10X Genomics. Chen-Shan Chin, Matthew Boitano, and Paul Peluso are employees of Pacific Biosciences. Paul Flicek is a member of the Scientific Advisory Board of Fabric Genomics, Inc. Evan E. Eichler is on the scientific advisory board (SAB) of DNAnexus, Inc., was a consultant for Kunming University of Science and Technology (KUST) as part of the 1000 China Talent Program (2014–2016), and was an SAB member of Pacific Biosciences, Inc. (2009–2013).

## Supplementary Material

Supplemental Material
