# Supplementary material for: Evaluation of GRCh38 and de novo haploid genome assemblies demonstrates the enduring quality of the reference assembly
Source: Genome Res. 2017 May;27(5):849–64. doi: 10.1101/gr.213611.116 (PMC5411779; doi:10.1101/gr.213611.116)
Supplement: Supplemental Material [file supp_27_5_849__index.html]

Evaluation of GRCh38 and de novo haploid genome assemblies demonstrates the enduring quality of the reference assembly — Supplemental Material 

# Evaluation of GRCh38 and de novo haploid genome assemblies demonstrates the enduring quality of the reference assembly

## Supplemental Material

- Supplemental\_Code.zip
- Supplemental\_GFF3\_S1.tar.gz
- Supplemental\_GFF3\_S2.tar.gz
- Supplemental\_Materials.pdf
- Supplemental\_VCF\_S1\_S2.zip
- Supplemental\_Worksheet\_S1.xlsx
- Supplemental\_Worksheet\_S2.xlsx
- Supplemental\_Worksheet\_S3.xlsx
- Supplemental\_Worksheet\_S4.xlsx
- Supplemental\_Worksheet\_S5.xlsx
- Supplemental\_Worksheet\_S6.xlsx
