# Supplementary material for: Evaluation of GRCh38 and de novo haploid genome assemblies demonstrates the enduring quality of the reference assembly
Source: Genome Res. 2017 May;27(5):849–64. doi: 10.1101/gr.213611.116 (PMC5411779; doi:10.1101/gr.213611.116)
Supplement: Supplemental Material [file supp_gr.213611.116_Supplemental_Materials.pdf]

Supplemental Material

Evaluation of GRCh38 and de novo haploid genome assemblies demonstrates the enduring quality of the reference assembly

Valerie A. Schneider[1], Tina Graves-Lindsay[2], Kerstin Howe[3], Nathan Bouk[1], Hsiu-Chuan Chen[1], Paul A. Kitts[1], Terence D. Murphy[1], Kim D. Pruitt[1], Françoise Thibaud-Nissen[1], Derek Albracht[2], Robert S. Fulton[2], Milinn Kremitzki[2], Vincent Magrini[2*], Chris Markovic[2], Sean McGrath[2], Karyn Meltz Steinberg[2], Kate Auger[3], William Chow[3], Joanna Collins[3], Glenn Harden[3], Timothy Hubbard[3#], Sarah Pelan[3], Jared T. Simpson[3%], Glen Threadgold[3], James Torrance[3], Jonathan M. Wood[3], Laura Clarke[4], Sergey Koren[5], Matthew Boitano[6], Paul Peluso[6], Heng Li[7], Chen-Shan Chin[6], Adam M. Phillippy[5], Richard Durbin[3], Richard K. Wilson[2], Paul Flicek[4], Evan E. Eichler[8,9], Deanna M. Church[1^]

Author Affiliations:
[1]National Center for Biotechnology Information, National Library of Medicine, National Institutes of Health, Bethesda, MD 20894, USA, [2]McDonnell Genome Institute at Washington University, St. Louis, MO 63018, USA, [3]Wellcome Trust Sanger Institute, Wellcome Genome Campus, Hinxton, Cambridge CB10 1SA, UK, [4]European Molecular Biology Laboratory, European Bioinformatics Institute, Wellcome Genome Campus, Hinxton, Cambridge, CB10 1SD, UK, [5]National Human Genome Research Institute, National Institutes of Health, Bethesda, MD 20892, USA, [6]Pacific Biosciences, Menlo Park, CA 94025, USA, [7]Broad Institute, Cambridge, MA 02142, USA, [8]Department of Genome Sciences, University of Washington School of Medicine, Seattle, WA 98195, USA, [9]Howard Hughes Medical Institute, University of Washington, Seattle, WA 98195, USA

Current Affiliations:
^10X Genomics, Pleasanton, CA 94566, USA
*Nationwide Children's Hospital, Columbus, OH 43205, USA
#King's College London, London, UK
%Ontario Institute for Cancer Research, Toronto, Ontario, Canada & Department of Computer Science, University of Toronto, Toronto, Ontario, Canada

This document contains:

Supplemental Notes
Supplemental Methods
Supplemental Figures S1-S6
Supplemental Tables S1-S11

Contents

Supplemental Notes 3
GRCh38 assembly updates 3
Assembly Gaps 3
Base updates 4
Impacts on read mapping 4
De novo assembly evaluation 4
Supplemental Methods 8
GRCh38 8
De novo assemblies 18
Supplemental Figures 23
Supplemental Tables 31
Supplemental References 38

Supplemental Notes

GRCh38 assembly updates

Assembly Gaps

Change in gap length is a more informative measure of reference assembly improvement than gap count. As noted in the main text, gap count increases when newly added sequence does not extend to gap edges, or if newly added sequence is itself gapped. Despite the increase in gap count in GRCh38, the gap length of the primary assembly unit (chromosomes, unlocalized and unplaced scaffolds) decreased by 11 Mb in GRCh38, exclusive of the additional 72 Mb of centromeric gaps in GRCh37 that were replaced by modeled sequence. However, it should be noted that change in gap length is still an imperfect metric for assessing reference assembly improvement, as the use of default gap lengths to represent biological gaps (10 kbp - 3 Mbp), unsequenced tiling path components (50 kbp) or unmeasured gaps between components or scaffolds (100 bp - 50 kbp) creates artifice and does not represent the actual amount of missing sequence in the reference genome assembly (International Human Genome Sequencing Consortium 2004). While there are approximately equal numbers of unspanned gaps with estimated and default lengths on the GRCh38 chromosomes, gaps using default sizing account for more than 80% of this gap length (Supplemental Table S2). Thus, additional analyses are required to determine the actual extent of sequence not represented in the human reference genome assembly and caution should be exercised when using gap length to assess newly identified gap-spanning sequences.

A comprehensive accounting of GRCh38 assembly gaps is provided in the following GenBank report:

ftp://ftp.ncbi.nlm.nih.gov/genomes/all/GCA/000/001/405/GCA_000001405.15_GRCh38/GCA_000001405.15_GRCh38_genomic.gaps.gz

Base updates

The coordinates of updated GRCh37 sites, along with their remapped locations in GRCh38 are provided as VCF files (Supplemental_VCF_S1.vcf, Supplemental_VCF_S2.vcf). In these VCFs, updated sites in the PAR regions on Chr Y have been assigned the variant ids corresponding to those in the following dbSNP VCF:

ftp://ftp.ncbi.nih.gov/snp/organisms/human_9606_b147_GRCh37p13/VCF/All_20160408_papu.vcf.gz

Impacts on read mapping

Of the 4.19% of NA24143 read pairs that map uniquely, albeit imperfectly, to the GRCh37 primary assembly in an unchanged assembly region and that move to a new location with a different underlying assembly component in GRCh38, approximately two-thirds have multiple alignments to GRC38. We also tracked the movements of reads belonging to these pairs and find that GRCh38 centromeric components are associated with 98% of these moved reads, consistent with the highly repetitive structure of these sequences (Supplemental Figure S3). Details of the read mapping analysis are provided in the Supplemental Methods.

De novo assembly evaluation

In an ongoing effort, the CHM1 and CHM13 de novo assemblies described herein are being evaluated to determine which might serve as the basis for further curation into a fully finished

platinum level assembly (http://genome.wustl.edu/projects/detail/reference-genomes-improvement/). The absence of allelic variation in these samples simplifies their assembly relative to diploid samples, as only repeat content remains to confound assembly construction. In addition, to better understand the impact join error thresholds and consensus algorithms used by the Celera Assembler have on assembly metrics, we generated a suite of CHM13 assemblies in which these parameters were systematically varied. We compared each of these to one another in addition to the FALCON CHM13 assembly, which is produced from the identical set of reads. Though we did generate additional CHM1 assemblies with Celera Assembler as part of this parameter evaluation, we present only the assembly that uses the same collection of input reads as the FALCON assembly in this manuscript, to avoid the influence of other factors, such as coverage or read chemistry might have on the comparison. The high N50s of the CHM1 and CHM13 assemblies are largely attributable to the haploid nature of the genomes, with the longest contig N50s being 26.9 Mbp and 19.4 Mbp, respectively. Consistent with the use of newer chemistry in the sequencing of the CHM1 sample, we find that contig N50s for CHM1 assemblies were higher than those of the CHM13 assemblies. Among Celera-based assemblies, those using the falcon_sense correction algorithm had slightly higher QV scores. For both samples, the FALCON-based assembly had the highest QV score, which most likely reflects a second round of Quivering that only these assemblies received.

Among the CHM13 assemblies, those with a higher allowed join error had larger N50s. We also observed substantial variability in the mean, median and maximum contig lengths, with all values higher in both FALCON-assembled genomes. In the Celera Assembler assemblies with the same error threshold, use of a more sensitive correction step is correlated with contig N50, but not mean or median contig length. Assemblies with a higher proportion of long contigs and a high N50 would be expected to offer the most sequence for reference gap curation efforts

(Pendleton et al. 2015; Chaisson et al. 2015; Shi et al. 2016). However, assemblies with higher N50s are also likely to have a higher proportion of erroneous joins.

While hybrid scaffolds generated from the CHM1 FALCON-based assembly exhibited slightly fewer discrepancies with respect to both the contigs and map scaffolds, all of the Celera Assembler CHM13 assemblies had fewer discrepancies than the FALCON-based CHM13 assembly. Among the Celera Assembler assemblies, a lower join error threshold correlates with fewer conflicts, as did use of falcon_sense (Berlin et al. 2015) as opposed to PBDAG-Con (Chin et al. 2013) for the correction step.

For both samples, the FALCON-based assemblies had a lower total area under the FRC compression-expansion curve. It is interesting to note that while the CHM1 assemblies are symmetrical for expansion and contraction, all CHM13 assemblies are skewed towards collapse. This may reflect the use of shorter reads generated with earlier sequencing chemistries in these samples. Among the CHM13 assemblies, there does not seem to be any correlation between correction algorithm or join error threshold and expansion, but the Celera Assembler assemblies generated with the lowest join error threshold appear the least collapsed, followed by the FALCON assembly and the Celera Assembler assemblies with the higher error threshold. When selecting an assembly to use for reference curation in segmentally duplicated, repetitive or complex genomic regions, these differences must be considered to ensure the most accurate representation.

In the FermiKit analyses of these assemblies, heterozygous variant call results for CHM13 were more tightly clustered and variable than for CHM1, but consistently show that Celera Assembler assemblies generated with the less permissive join threshold error exhibit the least collapse, while those with more permissive threshold exhibit the most, and the FALCON assembly falls in between. We also evaluated homozygous variant calls from alignment of FermiKit assemblies based on Illumina reads to the corresponding PacBio assemblies, as they are indicative of assembly errors, in an effort to establish overall quality (Supplemental Figure S6). These data

suggest a higher overall rate of insertion errors in the CHM1 assemblies than in the CHM13 assemblies. However, the CHM1 Illumina reads used as the input to the FermiKit assemblies were produced with older technologies and are therefore shorter and more error-prone than the CHM13 reads used for the same analyses, and may instead reflect deletion errors in the CHM1 FermiKit assemblies instead of differences in the PacBio assemblies of the two samples.

We used a number of other metrics to evaluate overall assembly quality. We also compared assembly contigs to BioNano optical maps we generated for the CHM1 and CHM13 assemblies. Scaffolding the contigs with the optical map data resulted in near doubling of the N50s and a 20-fold decrease in molecule number (Supplemental Table S8). For both the CHM1 and CHM13 samples, total numbers of indels and inversions were very low and comparable among assemblies in each set, demonstrating the overall high quality of these assemblies (Supplemental Table S9). We additionally performed paired end alignments of clones from a CHM1 derived BAC library (CHORI-17) to the CHM1 assemblies (Supplemental Table S10). We find that 96.64% (GCA_001307025.1) and 96.53% (GCA_001297185.1) of placements are unique and concordant, comparable to the 96.22% reported in an equivalent analysis of the CHM1_1.1 assembly (Steinberg et al. 2014). Based on these analyses, sequences from all of these assemblies appear to offer promise for use in gap closure or addition to the assembly as alternate loci.

Although the number of transcripts not aligning to the new assemblies is nearly double that of GRCh38, the new assemblies still offer representation for more than 99.5% of the transcripts in the input data set. Likewise the percentages of transcripts with <95% coverage or multiple best alignments (e.g. alignments split over >1 sequence) are also low, with >98% of all input transcripts aligning correctly. Transcript alignment issues are more common in the CHM13 Celera Assembler assemblies generated with the more conservative overlap error threshold than those with the more permissive threshold. While the FALCON-based assembly provides the superior overall gene representation for the CHM1 sample, three of the four Celera

Assembler assemblies provide better gene representation for CHM13. The numbers of dropped transcripts due to co-placement are slightly lower in the CHM13 assemblies with the more conservative join error threshold, consistent with the FRC^{bam} and FermiKit analyses indicating these assemblies exhibit slightly less collapse. For the CHM13 sample, we find that the proportion of frameshifted (FS) proteins common to all CHM13 assemblies does not differ substantially from the proportion of FS proteins unique to CHM1_1.1, suggesting that read quality does not make a large contribution to this metric. On the other hand, there are approximately 4x as many proteins with FS indels common to the two CHM1 PacBio assemblies as shared by both assemblies and CHM1_1.1, further demonstrating the influence of assembly method.

Consistent with prior multi-assembly evaluations, these analyses showed that no single assembly parameter or algorithm creates a best whole genome assembly across all metrics evaluated (Vezzi et al. 2012; Bradnam et al. 2013; Earl et al. 2011). More notably, they revealed that while de novo assemblies can now approach reference quality for some metrics, for others they still lag considerably behind GRCh38. Notably, the results show that assemblies with near reference quality length metrics may still lag with respect to gene representation, and that assembly methods contribute substantially to this assembly feature. In doing so, they illustrate that the use of sequence from such assemblies to close gaps or add to the reference assembly may not always be suitable.

Supplemental Methods

GRCh38

Assembly Updates

To ensure the continued high quality of the reference assembly, we adhered to operating procedures developed for GRC assembly updates for the sequencing and finishing of components, selection of previously sequenced clone and WGS components identified in the INSDC, and addition of components to the assembly tiling path, including the evaluation of component overlaps (Church et al. 2011) (https://www.ncbi.nlm.nih.gov/projects/genome/assembly/grc/info/index.shtml).

Transcript Evaluation of Assemblies

We obtained the FASTA for GENCODE 23 transcripts from: ftp://ftp.sanger.ac.uk/pub/gencode/Gencode_human/release_23/gencode.v23.chr_patch_hapl_scaff.transcripts.fa.gz. From these files we extracted the "basic" transcripts using the sequence identifiers from ftp://ftp.sanger.ac.uk/pub/gencode/Gencode_human/release_23/gencode.v23.chr_patch_hapl_scaff.basic.annotation.gff3.gz. We extracted the sequence identifiers for human transcripts from the RefSeq 71 release from ftp://ftp.ncbi.nlm.nih.gov/refseq/release/release-catalog/archive/RefSeq-release71.catalog.gz.

"Known" human RefSeq transcripts (prefix NM or NR) were queried from the NCBI RefTrack database on November 20, 2015.

GENCODE 23 and RefSeq 71 sequences were aligned to GRCh37 (GCF_000001405.13) and GRCh38 (GCF_000001405.26). "Known" transcripts were aligned to GRCh38 (GCF_000001405.26), CHM1_1.1 (GCF_000306695.2) and the collection of CHM1 and CHM13 assemblies (GCA_001297185.1, GCA_001307025.1, GCA_000983475.1, GCA_000983465.1, GCA_001015355.1, GCA_001015385.3 and GCA_000983455.2).

Centromere Sequence Additions

Intact sequences from the LinearCen1.1 (normalized) assembly (GCA_000442335.2) were incorporated into the corresponding chromosomes of the reference assembly. We made the following exceptions to allow for the reordering of components as determined by the GRC or replacement of WGS with clone-based sequence.

CM000664.2: Excluded LinearCen1.1 scaffold GJ211862.1, comprised solely of HuRef WGS contigs. It was deemed redundant to AC026273.7, a BAC clone that was contiguous with the existing assembly tiling path.

CM000671.2: Excluded components ABBA01045076.1 and ABBA01045076.1 from LinearCen1.1 scaffold GJ211880.1, comprised solely of HuRef WGS contigs. They were deemed redundant with FP325717.1, a BAC clone contiguous with the existing assembly tiling path. The order of the retained WGS contigs from GJ211880.1 was reversed to preserve the ordering with respect to FP325717.1.

CM000672.2: The HuRef WGS contigs comprising LinearCen1.1 scaffold GJ211931.1, had their path reversed and were moved outside the centromere region to maintain continuity with AC127389.2, a clone component in the existing assembly tiling path.

CM000682.2: Inserted clone component AL837517.14 into the centromere region to close the gap between HuRef WGS contigs ABBA01015878.1 and ABBA01015879.1 in LinearCen1.1 scaffold GJ211996.1. Replaced redundant LinearCen1.1 scaffold GJ211966.1 component ABBA01031673.1 with clone component AC011850.12.

Non-satellite HuRef centromere-associated contigs were added to the assembly as unplaced scaffolds if they did not have at least two of the following forms of evidence linking them to a specific chromosome:

1. Paired read support from the HuRef genome (GCA_000002125.2): where at least two paired reads were observed to link a given alpha satellite reference model and an unmapped contig (as provided by localizing the paired read to the assembly data generated by the assembly submitter).

2. Evidence for assignment to a particular chromosome using available flow-sorted chromosome data and unique *k*-mer mapping, intersecting published datasets from (Altemose et al. 2014).
3. Evidence for assignment using published admixture studies from (Genovese et al. 2013).

The collection of unplaced centromere-associated scaffolds includes local names HSCHRUN_RANDOM_100 to HSCHRUN_RANDOM_204.

GRCh38 also includes modeled representation for a heterochromatic region on Chromosome 7. The locations of all modeled GRCh38 regions are provided as annotations on each INSDC chromosome record, at the GRC website (https://www.ncbi.nlm.nih.gov/projects/genome/assembly/grc/human/) and are also defined in the regions file available for download from the GenBank FTP site (ftp://ftp.ncbi.nlm.nih.gov/genomes/all/GCA/000/001/405/GCA_000001405.15_GRCh38/GCA_000001405.15_GRCh38_assembly_regions.txt).

ClinVar analyses

We used the NCBI Genome Remapping Service, to remap variants from GRCh37 to GRCh38. We successfully remapped all variants in this set (Kitts et al. 2016). The list of ClinVar variants that remapped ambiguously in the GRCh38 primary assembly is provided in Supplemental Worksheet S4. The column definitions for this report, described comprehensively in https://www.ncbi.nlm.nih.gov/genome/tools/remap/docs/whatis, are as follows:

1. #feat_name: user-supplied feature name. If no feature name is supplied, a name is calculated using the line number in the file or the location. For features with multiple intervals (e.g. transcripts), this field will be common to each interval.
2. source_int: The number of intervals in the source feature (useful for tracking features with multiple intervals, like transcripts). For single-interval features, the value is always 1.

3. mapped_int: the number of mapped intervals in the remapped file from the source interval. Values >1 indicate a fragmented mapping.
4. source_id: sequence identifier the feature maps to in the source file.
5. mapped_id: sequence identifier the features maps to on the target assembly.
6. source_length: length of the interval on the source assembly.
7. mapped_length: length of the interval on the target assembly.
8. source_start: first base of the interval on the source assembly.
9. source_stop: last base of the interval on the source assembly.
10. source_strand: strand the interval is annotated on in the source assembly.
11. source_sub_start: first base of source sub-interval that was mapped (used only if entire source interval does not remap and the front edge of the source interval does not map).
12. source_sub_stop: last base of source sub-interval that was mapped (used only if entire source interval does not remap and the back edge of the source interval does not map).
13. mapped_start: first base of remapped interval.
14. mapped_stop: last base of remapped interval.
15. mapped_strand: strand of remapped base.
16. coverage: This is calculated by taking the ratio of the mapped_length to the source_length. If coverage =1 the remapped and source interval are identical. A coverage score of less than 1 indicates a deletion in the target assembly and a score of greater than 1 indicates an insertion in the target assembly.
17. recip: Two possible values are in this column. First Pass means the remapping is based on the 'First Pass' or reciprocal-best-hit alignments. 'Second Pass' means the remapping is based on the non-reciprocal-best-hit alignments.
18. asm_unit: The assembly unit to which the mapped_id belongs. For more information on assembly units, see: https://www.ncbi.nlm.nih.gov/assembly/model/.

Base Updates

Identification of candidate erroneous bases

We identified candidate erroneous bases by searching for regions of the GRCh37 reference sequence that were discordant with reads from the 1000 Genomes Project. To reduce the impact of alignment errors in repetitive regions we used a conservative alignment-free strategy based on counting *k*-mers (here *k*=61). We used a two-stage filtering procedure to find all 61-mers in the GRCh37.p9 sequence that were seen fewer than 5 times in the 1000 Genomes reads. In the first stage we screened each 61-mer in the reference sequence against high-coverage sequence reads from the sample NA12878 using the FM-index implemented in SGA (Simpson and Durbin 2012). The reference 61-mers seen fewer than 5 times in NA12878 were loaded into a hash table. We then iterated over the 1000 Genomes reads to directly count the number of times these 61-mers were seen. After eliminating the 61-mers seen at least 5 times, a set of 6.7 million 61-mers remained. As a single incorrect base will generate up to 61 unique 61-mers, we grouped adjacent 61-mers into discordant regions. This procedure generated approximately 50,000 candidate regions. These regions were intersected with SNVs reported to have a reference allele frequency of < 0.05 according to 1000 Genomes phase 1 data, and indels with an MAF=0 that fall within the phase 1 accessibility mask, resulting in 13,887 single bps and 2,424 indels in need of confirmation. We manually reviewed the subset of indels with less than 101 bp distance between each other, leading to a revised collection of 2,372 indels. From this subset, we further analyzed 75 putative indels on Chr 20, confirming them as monomorphic in the available experimental 1000 Genomes phase 3 data or with no representation in reads from the RP11 sample.

Additional candidate SNV bases were drawn from the following files, generated as part of analyses on the 1000 Genomes phase 1 dataset, and added to the *k*-mer derived set:

ftp://ftp.1000genomes.ebi.ac.uk/vol1/ftp/technical/working/20120803_grc_cdna_analysis/20120830_high_confidence_rare_mono_sites_for_change.txt.gz

ftp://ftp.1000genomes.ebi.ac.uk/vol1/ftp/technical/working/20120803_grc_cdna_analysis/20121025_strict_rare_and_mono_sites_monomorphic.txt.gz

ftp://ftp.1000genomes.ebi.ac.uk/vol1/ftp/technical/working/20120803_grc_cdna_analysis/20121206_overlapping_pseudogenes.txt.gz

The final set of final set of candidates additionally included bases drawn directly from community requests reported in the GRC issue tracking system.

Details for WGS mini-contig generation

General rules:

1. Build mini-contigs from reads overlapping and surrounding the base in question (roughly ±200 flanking bp)
2. Minimum contig size = 50 bp
3. Only readsets with at least 1 read that has base quality 50 at the target base are eligible for use
4. Minimum coverage at any base in built contig = 4
5. Exclude any SOLiD readsets

cortex_con runs:

1. Define the most-appropriate sequence source to use for contig building at a target base
    a. If target base is in an RP11 assembly component, use reads from SRR834589
    b. If target base is in an assembly component from another library, determine whether there are samples in 1000 Genomes phase 1 project that are a population match
        i. The population origin for assembly components was as defined in the supplemental data of Green et al. (Green et al. 2010): RP11=NA; RP1,3,4,5 (same sample)=CEU; CTA,CTB (same sample)=CEU; CTC=CEU; CTD=JPT.

2. Perform the following hierarchical series of cortex_con runs, with a validation step between each (see below):
    a. Input: Individual high_coverage samples.
        i. If component individual is known, use only readsets from that sample
        ii. If component population is known or there is no success with the known individual, use only readsets from samples belonging to that population
        iii. If component population is not known or there is no success with the known population, randomly cycle though all high coverage samples
        iv. If there is success with high_coverage data, do not attempt further cortex_con runs
    b. Input: Grouped low_coverage/exome samples
        i. If component individual is known, use only grouped readsets from that sample
        ii. If component population is known, only use grouped readsets from samples belonging to that population
        iii. If component population is not known or there is no success with the known population, cycle randomly though populations, grouping 50 readsets per population

Validation

1. After a successful cortex run, align output to a defined window in the vicinity of the target base
2. Filter for contigs whose alignments have only a single mismatch at the base in question
3. Check that the nucleotide at mis-matched target base is the expected nucleotide.
4. If no contigs pass validation, perform the next cortex_con run.

The 5% of target bases at which we failed to generate a mini-contig in the initial run underwent subsequent alignments (i.e. allow max of two mismatches, and so on) to see how many more would be addressed if we also modified surrounding bases. In a subset number of cases we elected to update nearby bases in addition to the target base, if review of the 1000 Genomes phase 1 data revealed those other bases to be in LD with one another.

There are also instances in which target bases lie within ~200 bp of one another. In such cases, we flagged target clusters up-front and built/validated for contigs containing the set of base updates. We reviewed clustered targets before contig building to distinguish whether they result from a bad assembly component (i.e. multiple sequencing errors) or highly variant genomic regions at which variant calls might be suspect, and discarded the latter.

Alignment of Illumina reads from Ashkenazim sample

- Sample: NA24143
    - BioSample ID: SAMN03283346 (Ashkenazi trio mother)
- Reads: 2x150bp HiSeq2500 PCR-free, paired
    - Insert size: ~564 bp end-to-end (including the reads at either end)
    - ftp://ftp-trace.ncbi.nlm.nih.gov/giab/ftp/data/ftp/technical/NISTAshkenazimTrio/HG-004_Homogeneity-14572558/HG004_HiSeq300x_fastq/140818_D00360_0046_AHA5R5ADXX/
    - Downsampling: The original 300x fastq data were downsampled to ~10x coverage
        - Only reads where all bases qual>20 (ASCII char >5) were accepted
        - Alignment input: 242M reads (121M pairs)
- Aligner: BWA-MEM 0.7.12-r1044 with post-processing for alternate loci
    - Parameters: default, except -I 550,150,1200,1

- Target assemblies (all include Chr EBV and Chr MT, and only full includes alt loci):
  - GRCh37pme: ftp://ftp.ncbi.nlm.nih.gov/genomes/archive/old_genbank/Eukaryotes/vertebrates_mammals/Homo_sapiens/GRCh37.p13/seqs_for_alignment_pipelines/GCA_000001405.14_GRCh37.p13_no_alt_analysis_set.fna.gz
  - GRCh38pme: ftp://ftp.ncbi.nlm.nih.gov/genomes/all/GCA/000/001/405/GCA_000001405.15_GRCh38/seqs_for_alignment_pipelines.ucsc_ids/GCA_000001405.15_GRCh38_no_alt_analysis_set.fna.gz
  - GRCh38full: ftp://ftp.ncbi.nlm.nih.gov/genomes/all/GCA/000/001/405/GCA_000001405.15_GRCh38/seqs_for_alignment_pipelines.ucsc_ids/GCA_000001405.15_GRCh38_full_analysis_set.fna.gz

Unmapped Read Analysis

We used samtools to identify the set of unmapped reads and to count mapped reads in GRCh37pme, GRCh38pme and GRCh38full:

samtools view -f12 (read and mate unmapped)

samtools view -f 0x4 -F 0x8 (singleton unmapped)

samtools view -f 0x40 -F 0x4 (read 1 mapped)

samtools view -f 0x80 -F 0x4 (read 2 mapped)

Aligned Read Movement Analysis

Read movements were calculated with a custom C++ script on the BWA-MEM alignments of the Ashkenazi Illumina data. Unchanged assembly regions were defined as those at which the assembly component was identical in GRCh37 or GRCh38, or at which a component update

between assembly versions only involved sequence not used in either assembly. We evaluated the movement of reads with unique, but imperfect, alignments in these regions on GRCh37pme. We considered a read “moved” if its primary alignment in GRCh38pme was on a different assembly component than its alignment in GRCh37pme.

We excluded reads whose alignments to GRCh37pme had the following characteristics:

- SA flag (supplementary flag) (2048 or 0x800)
- XA flag (secondary flag) (256 or 0x100)
- >1 primary alignment (e.g. reads that have >1 row and neither row has SA or XA flag)
- no flags set (e.g. a mapped single segment) (0)
- unmapped flag (4 or 0x4)
- One or both reads in pair is outside unchanged region in GRCh37pme

For read-pairs comprised of the remaining reads, we evaluated the GRCh37pme and GRCh38pme mapping locations of the subset of reads with “imperfect” alignments, defined as those meeting one or more of the following criteria:

- reads not properly paired
- 1 or both reads do not have NM:i:0
- 1 or both reads do not have MD:Z:148

De novo assemblies

Assembly Methods

A summary of the de novo assembly methods is provided in Supplemental Table S3. Additional details are provided below and in Supplemental Figures S4 and S5.

For assemblies generated by Celera Assembler, raw sequences were overlapped using MHAP and corrected read consensus was generated with either falcon_sense (Berlin et al. 2015) or PBDAG-Con (Chin et al. 2013) and assembled at 2.5% error or 5% error to evaluate the effects of read correction algorithms and overlap error rate on assembly quality and continuity.

Algorithm-related parameters for the Celera Assembler assemblies are as follows:

CHM1_CA_P6 (GCA_001307025.1): ovlerr=2.5%; minovl=100; falconsense

CHM13_CA1 (GCA_000983465.1): ovlerr=5.0%; minovl=100; falconsense

CHM13_CA2 (GCA_001015355.1): ovlerr=5.0%; minovl=100; pbdagcon

CHM13_CA3 (GCA_000983475.1): ovlerr=2.5%; minovl=100; falconsense

CHM13_CA4 (GCA_001015385.3): ovlerr=2.5%; minovl=100; pbdagcon

FALCON-generated assemblies underwent additional steps before the final polishing step with the Quiver algorithm to achieve a more comprehensive genome representation than would normally be achieved. The FALCON assembler uses the overlap count of each error-corrected read to infer whether the reads end in high copy repeats, and may remove such reads from the initial assembly step to reduce graph complexity. Additionally, the default assembler output does not include the first 5'-reads. In the modification to the pipeline, if the read on the 5'-end of a contig is not used by another contig, the read sequence is prepended to the contig. Reads that have >80 overlaps on either the 5'- or 3'-ends are filtered out. These reads that are initially filtered out are then collected and re-assembled separately with a higher overlap-count threshold (100,000 overlaps). These sub-assemblies from such highly repetitive sequences may have more mis-assemblies, but they are useful for applications in which it is desirable that all genome content should be captured (e.g. use as decoy sequences for reducing remapping errors).

Algorithm-related parameters for the FALCON assemblies are as follows:

```
 CHM1 config file:

# The length cutoff used for seed reads used for initial mapping

length_cutoff = 15000

# The length cutoff used for seed reads used for pre-assembly
```

```
length_cutoff_pr = 15000

pa_HPCdaligner_option =  -v -dal128 -t16 -H15000 -e0.75 -M24 -l4800 -k18 -
h480 -w8 -s100
ovlp_HPCdaligner_option =  -v -dal128  -M24 -k24 -h1024 -e.96 -l2500 -s100 -
H15000

pa_DBsplit_option = -a -x500 -s400
ovlp_DBsplit_option = -s400

falcon_sense_option = --output_multi --output_dformat --min_idt 0.70 --
min_cov 4 --max_n_read 400 --n_core 12
falcon_sense_skip_contained = False

overlap_filtering_setting = --max_diff 40 --max_cov 80 --min_cov 2 --n_core
12
```

CHM13 config file:

```
[General]
# The length cutoff used for seed reads used for initial mapping
length_cutoff = 10000
# The length cutoff used for seed reads used for pre-assembly
length_cutoff_pr = 10000

pa_HPCdaligner_option =  -v -dal128 -t16 -H10000 -e0.75 -M24 -l3200 -k18 -
```

```
h480 -w8 -s100
ovlp_HPCdaligner_option =  -v -dal128  -M24 -k24 -h1024 -e.96 -l1800 -s100 -
H10000

pa_DBsplit_option = -x500 -s400
ovlp_DBsplit_option = -x500 -s400

falcon_sense_option = --output_multi --output_dformat --min_idt 0.70 --
min_cov 4 --max_n_read 200 --n_core 8
falcon_sense_skip_contained = False

overlap_filtering_setting = --max_diff 40 --max_cov 80 --min_cov 2 --n_core
12
```

Assembly Evaluation Methods

Feature Response Curves were generated with FRC^{bam} (https://github.com/vezzi/FRC_align), using Illumina PE data and a genome size of 3.1 Gbp to evaluate the assembly and identify suspect assembly regions associated with high or low coverage and stretched and compressed read-pairs (Vezzi et al. 2012). Illumina sequences were mapped to each assembly using BWA-MEM (Li 2013).

The FermiKit pipeline was used to generate de novo assemblies of Illumina reads and perform variant calling on the various PacBio assemblies (Li 2015). Variant calls are available from: ftp://ftp.broadinstitute.org/aseval.

To define QV scores, reads were mapped with BWA-MEM (Li 2013) and variants were called with FreeBayes (https://github.com/ekg/freebayes) (Garrison and Marth 2012). If the majority of Illumina reads disagreed with an assembly base, the assembly was considered incorrect and a variant was called. A minimum of 3 reads was required to make any call. The QV was computed using the standard Phred formula, (-10 log_base10(P)) with P = total count of errors with at least 3-fold coverage divided by total bases with at least 3-fold coverage (Ewing and Green 1998). Any detected variant supported by a majority of the Illumina reads was considered an error in the assemblies.

SV Detection with BioNano Maps

SV calls on most assemblies were made with the following version of the BioNano software:

- Pipeline Version: $Id: SVModule.py 3835 2015-05-21 19:28:48Z wandrews $
- RefAligner Version: SVNversion=3827

The calls on GCA_000983455.2 and GCA_001015385.3 were made with an updated version:

- Pipeline Version: $Id: PairwiseModule.py 4125 2015-09-19 00:31:05Z wandrews $
- RefAligner Version: SVNversion=4287

Supplemental Figures

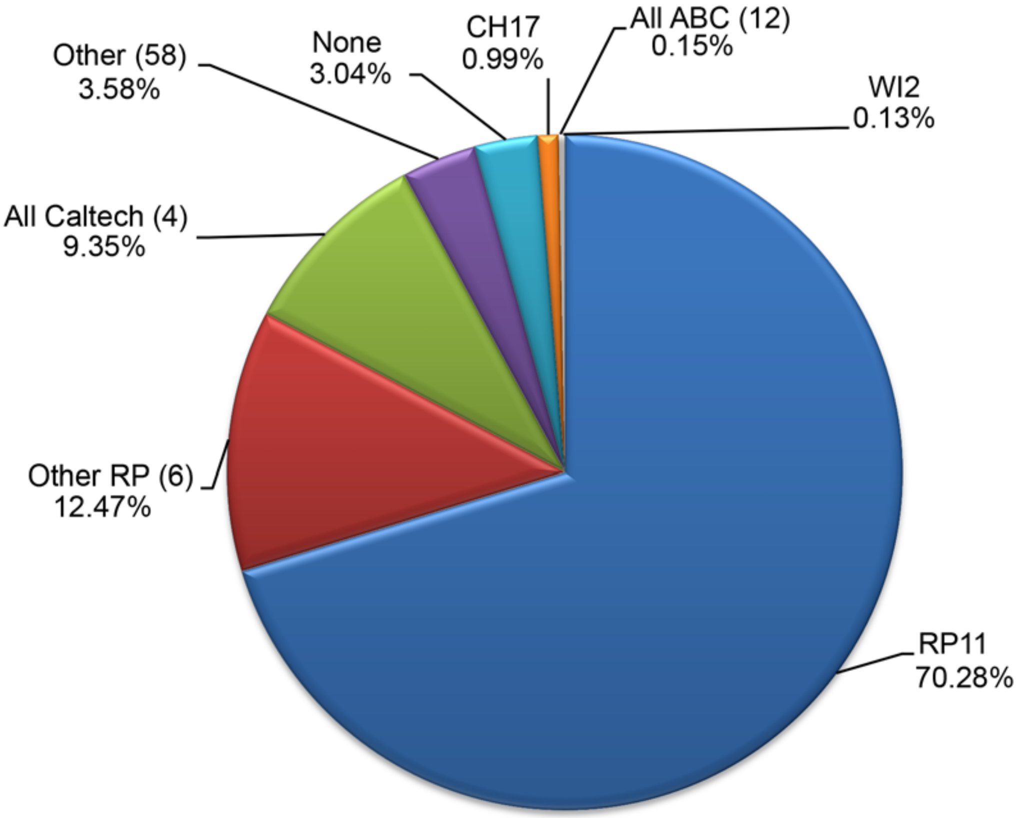

Supplemental Figure S1

Supplemental Figure S1|Genomic library composition of GRCh38 primary assembly. The proportion of ungapped sequence contributed by each genome library is shown. In some cases, genomic clone libraries have been grouped by creator (RP: Roswell Park Cancer Institute, Caltech, ABC: Agencourt Biosciences). The count of libraries in each groups is shown. None = WGS or PCR sequence. Clone libraries are described in the NCBI Clone DB (Schneider et al. 2013).

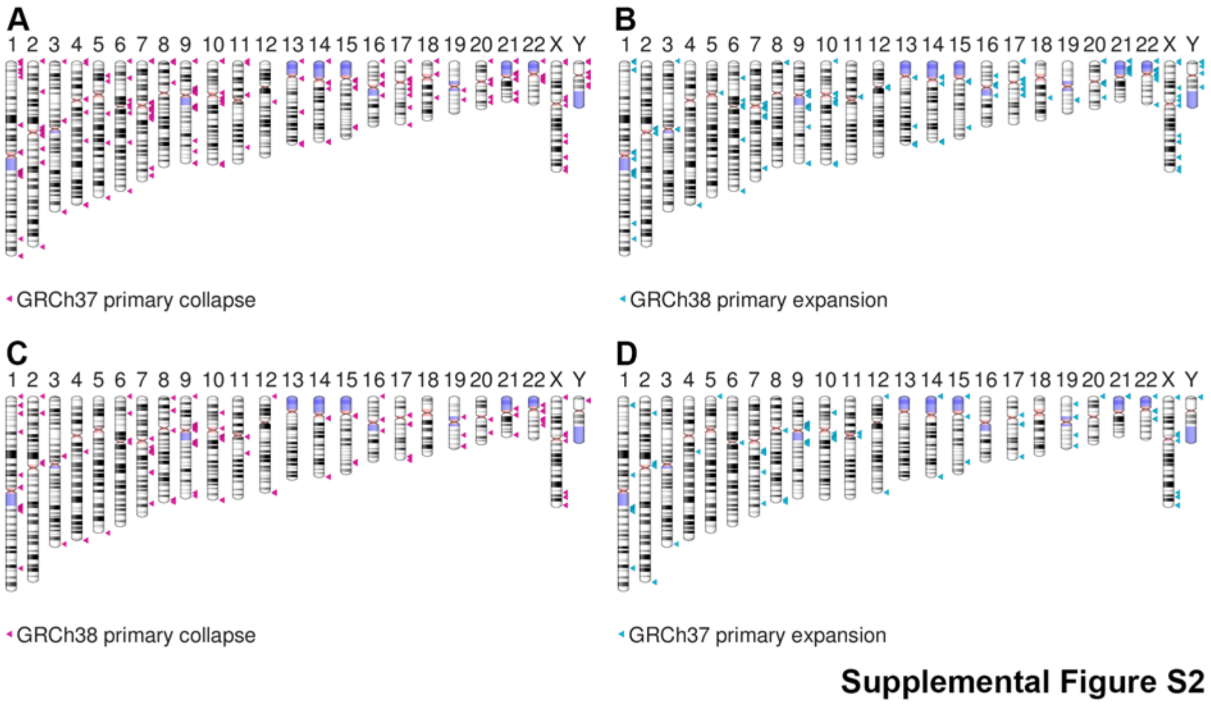

Supplemental Figure S2|Collapse and expansion in GRCh37 and GRCh38. (A,B) Ideograms showing the location of collapsed sequence in GRCh37 and expanded sequence in GRCh38, based on the alignments of their primary assembly units. The sites are largely overlapping (C,D) Ideograms showing the location of collapsed sequence in GRCh38 and expanded sequence in GRCh37, based on the alignments of their primary assembly units. There is also substantial overlap among these sites.

Supplemental Figure S3

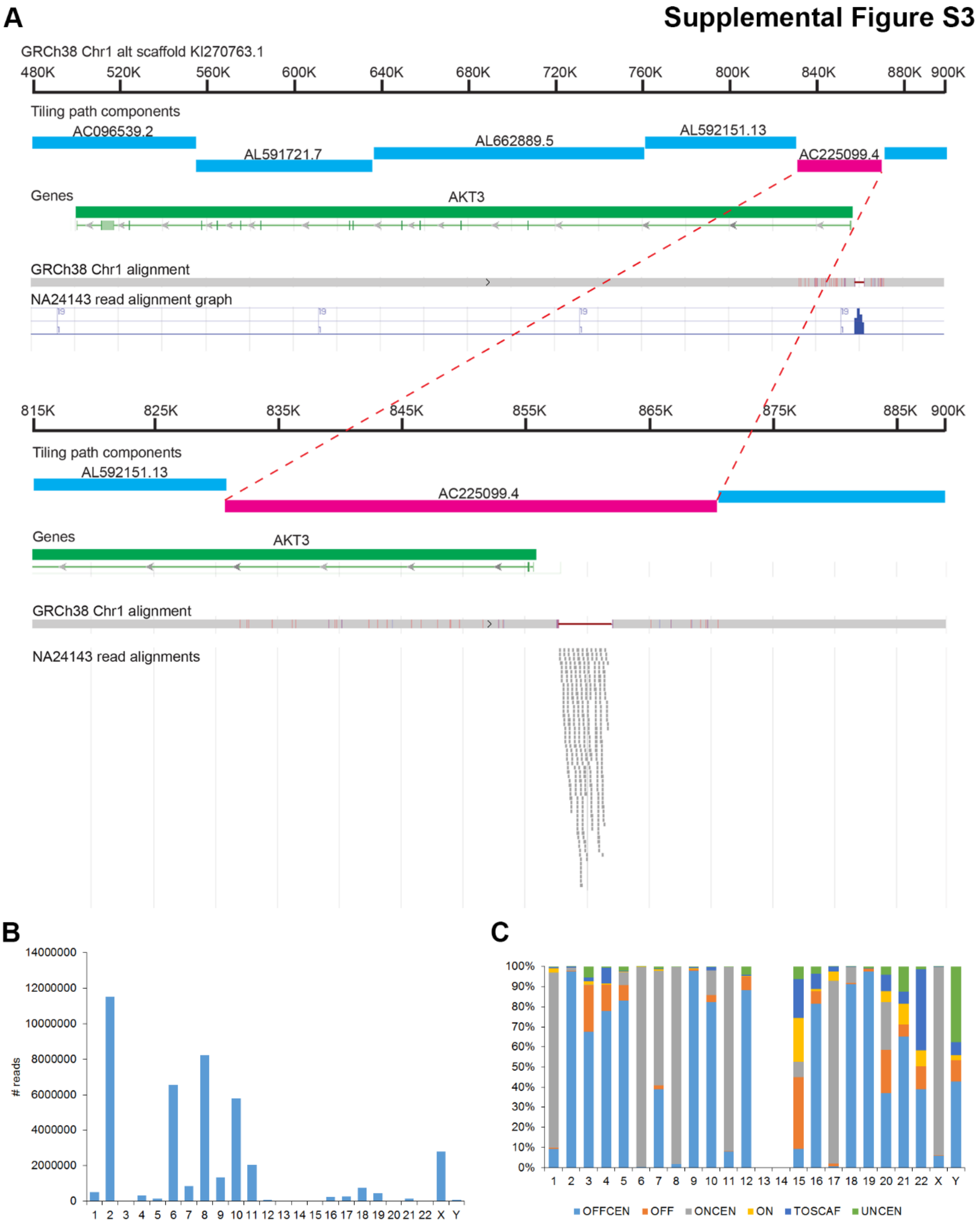

Supplemental Figure S3|NA24143 read alignments to GRCh38. (A) Schematic showing the alignment of a subset of reads unmapped on the GRCh38 primary assembly unit to the GRCh38 full assembly. Reads align to the GRCh38 alternate loci scaffold KI270673.1 at the position of an insertion (thin red line) relative to the corresponding chromosome region. (B) Graph showing counts of reads uniquely mapped to unchanged regions of GRCh37 that map non-uniquely to non-equivalent locations in GRCh38. (C) Chart describing the GRCh38 distribution of reads from (B), categorized by sequence location (same or different chromosome/scaffold) and sequence type (centromeric vs. non-centromeric). OFFCEN = movement to centromeric sequence on different chromosome; OFF = moment to non-centromeric sequence on different chromosome; ONCEN = movement to centromeric sequence on same chromosome; ON = movement to non-centromeric sequence on same chromosome; TOSCAF = movement to a non-centromeric unlocalized or unplaced scaffold; UNCEN = movement to an unplaced scaffold containing centromere-associated sequence.

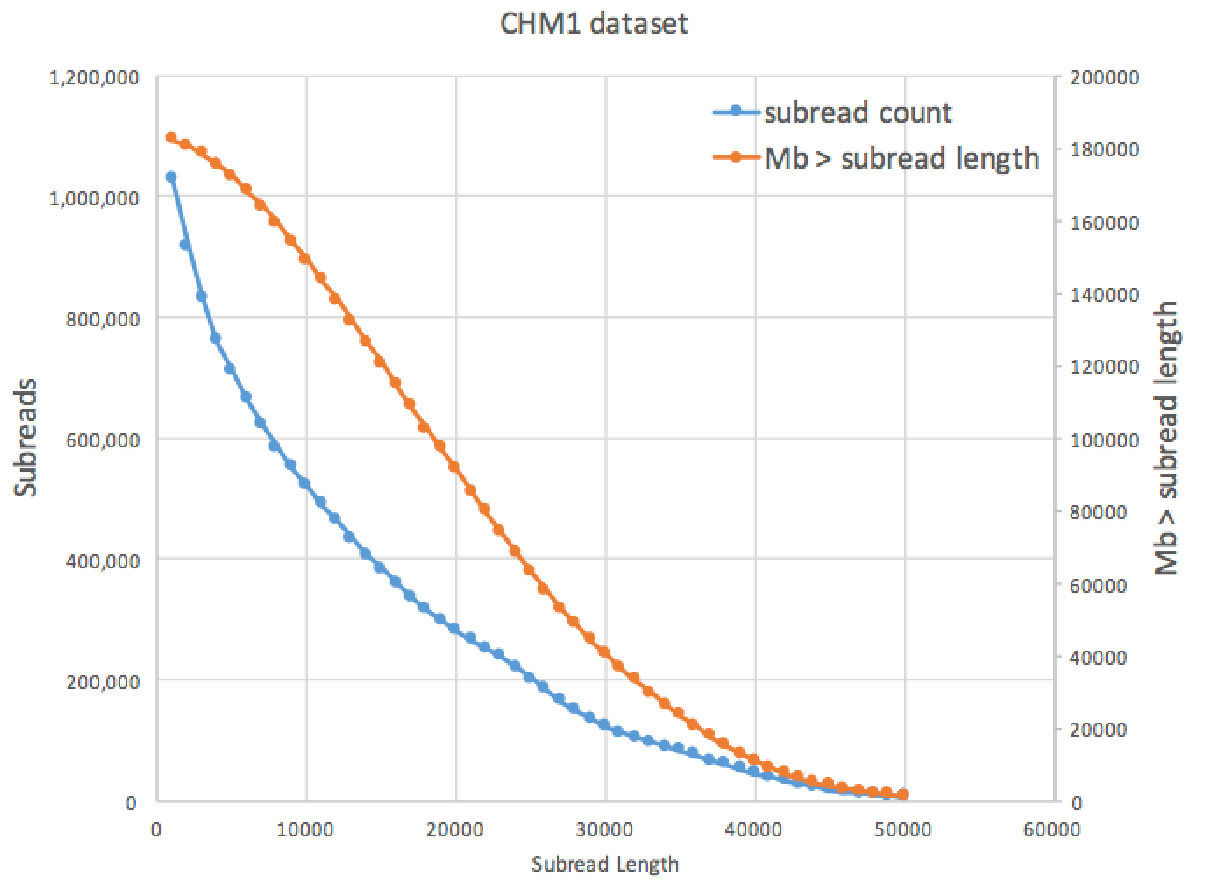

Supplemental Figure S4

Supplemental Figure S4|PacBio subread length distributions from CHM1 sample SRP044331. The blue curve provides the count of sub-reads (left y-axis) for a given length (x-axis). The orange curve represents the amount of sequenced bases (right y-axis) greater than a given subread length (x-axis). Sequencing was done on the PacBio RS II with P6-C4 chemistry.

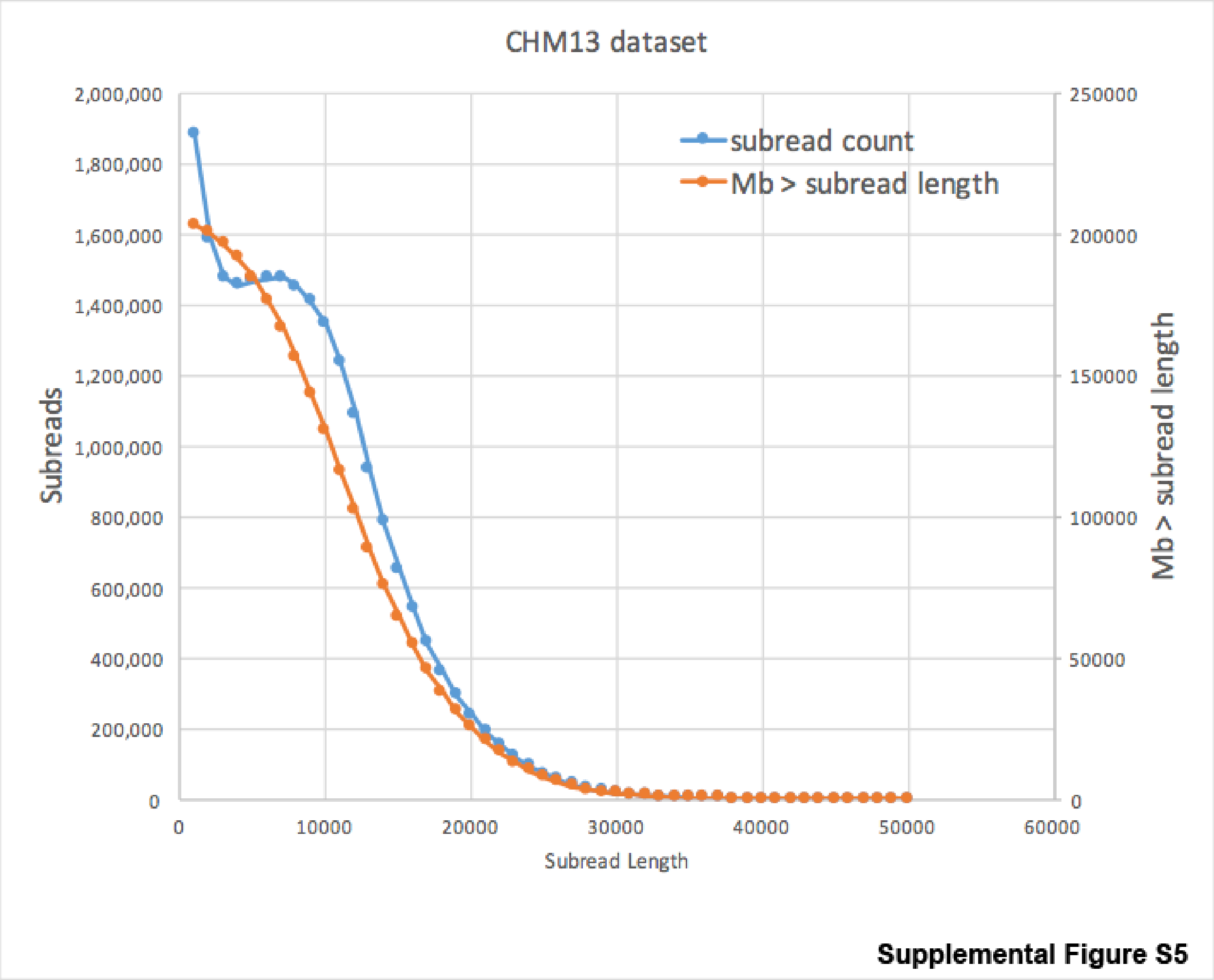

Supplemental Figure S5|PacBio subread length distributions from CHM13 sample SRP051383. The blue curve provides the count of sub-reads (left y-axis) for a given length (x-axis). The orange curve represents the amount of sequenced bases (right y-axis) greater than a given subread length (x-axis). Sequencing was done on the PacBio RS II with P5-C3 and P6-C4 chemistries.

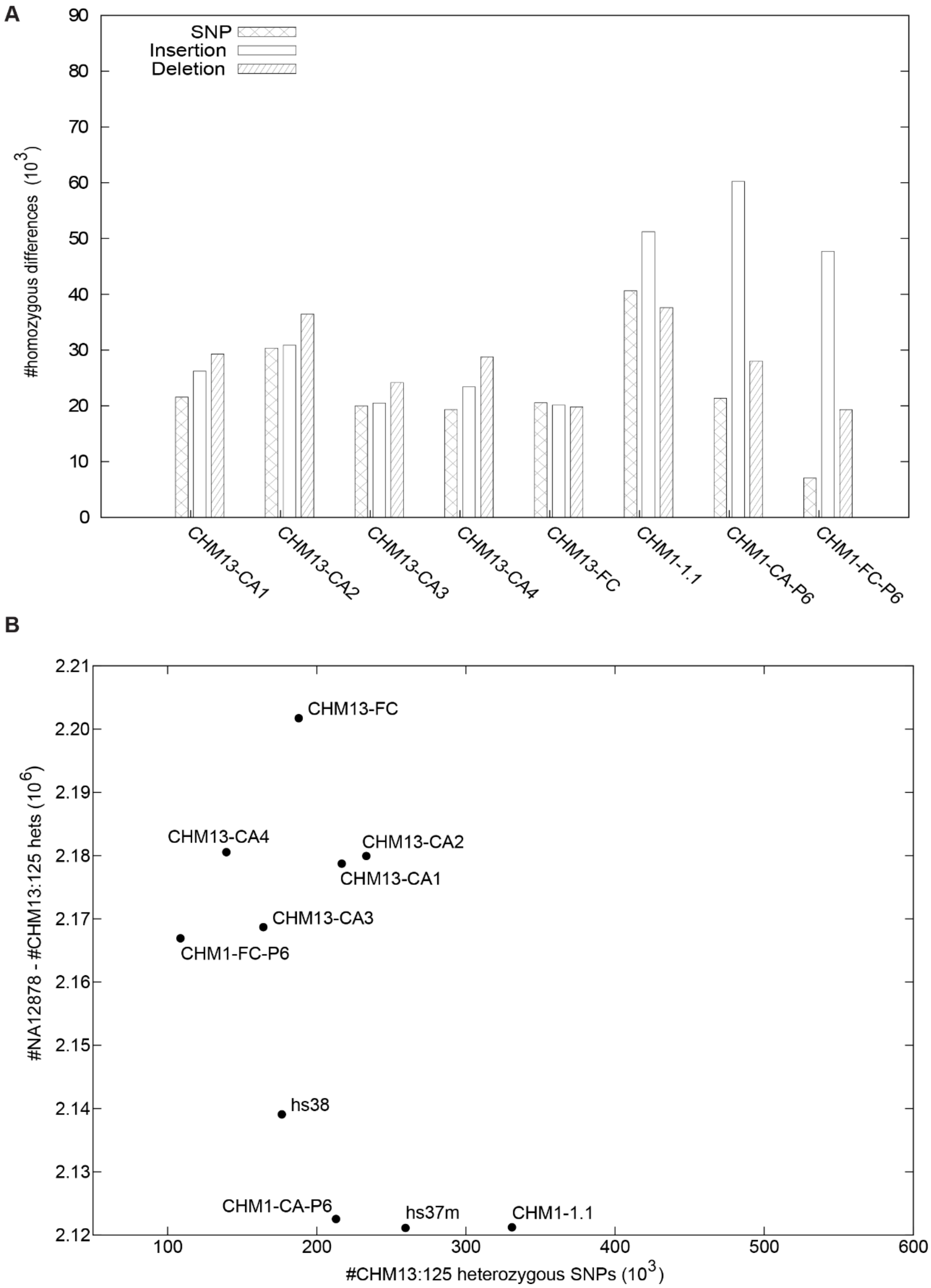

Supplemental Figure S6

Supplemental Figure S6|Additional evaluation of CHM1 and CHM13 assemblies. (A) Homozygous SNPs called on the CHM1 and CHM13 de novo assemblies using CHM1 and CHM13 aligned FermiKit assemblies. (B) Heterozygous SNPs called on the CHM1 and CHM13 de novo assemblies, CHM1_1.1 and GRCh38 using NA12878 and CHM13 aligned FermiKit assemblies. The x-axis represents potential false positives, while the y-axis measures potential true positives; optimal assemblies appear in the upper left of the plot.

Supplemental Tables

Supplemental Table S1. Sequence types flanking GRCh38 assembly gaps

	GRCh38		GRCh37	
**Sequence Types of Gap Flanks**	**Unspanned Chromosome Gaps***	**Spanned Chromosome Gaps***	**Unspanned Chromosome Gaps***	**Spanned Chromosome Gaps***
**WGS-WGS**	15	113	6	2
**WGS-HTG**	13	17	4	2
**HTG-HTG**	83	87	129	82

***Exclusive of gaps within centromere regions or abutting biological gaps**

Supplemental Table S2. Sizing methods for GRCh38 Primary Assembly Unit gaps

Gap Type*	Gap Sizing^	Gap Location	Gap Count	Length (bp)
**Biological**	default	chromosome	64	137,377,000
**Unspanned**	estimated	chromosome	143	1,820,364
**Unspanned**	default	chromosome	142	8,400,000
**Spanned**	estimated	chromosome	221	1,013,364
**Spanned**	default	chromosome	213	2,019,708
**Spanned**	estimated	unlocalized or unplaced scaffold	86	192,243
**Spanned**	default	unlocalized or unplaced scaffold	6	300,000

***Biological gaps include gaps defined as telomere, short arm and heterochromatin in AGP v2.0 and TPF files. Unspanned gaps are known as AGP v2.0 “contig” gaps or TPF “type-3” gaps. Spanned gaps are known as AGP v2.0 “scaffold” gaps and include TPF “type-2” gaps, as well as gaps >10 bp within assembly components.**

^Estimated gaps have lengths that were approximated according to one of the following methods: paired ends, align genus, optical map or fingerprint. Default gaps have lengths that reflect the use of default sizes.

Supplemental Table S3. Statistics for NA24143 alignments on GRCh37 and GRCh38

	GRCh37pme*	GRCh38pme+	GRCh38full^
**Total Reads (242,367,770)**			
**Aligned 1 reads**	121,092,818	121,149,469	121,157,632
**Aligned 2 reads**	121,092,098	121,149,105	121,157,274
**Read and mate unmapped**	138,612	46,060	32,754
**Singleton unmapped**	44,242	23,136	20,110
**Captured from GRCh37pme unmapped**	NA	117,617	ND
**Captured from GRCh38pme unmapped**	NA	NA	16,407
**Read-pairs in GRCh37pme unchanged regions with unique, imperfect alignments**	29,719,121	NA	NA
**Improperly paired**	76,827	NA	NA
**Imperfectly aligned end(s)**	29,375,537	NA	NA
**Improperly paired & imperfectly aligned**	266,757	NA	NA
**Subset of those read pairs that moved location in GRCh38pme**	NA	1,245,281	
**Unique in GRCh38pme**	NA	452,564	NA
**Perfect alignment**	NA	83,499	NA
**Improperly paired**	NA	5,166	NA
**Imperfectly aligned end(s)**	NA	315,978	NA
**Improperly paired & imperfectly aligned**	NA	47,954	NA
**Multiple in GRCh38pme**	NA	791,760	NA
**No longer paired in GRCh38pme**	NA	936	NA
**Both reads unmapped in GRCh38pme**	NA	21	NA

* GRCh37 primary assembly unit (excludes alt loci), mitochondria and EBV
+ GRCh38 primary assembly unit (excludes alt loci), mitochondria and EBV
^ GRCh38 full assembly (includes alt loci), mitochondria and EBV

Supplemental Table S4. Counts for NA24143 read movements (unique GRCh38 mappings)

Chromosome	OFFCEN	OFF	ONCEN	ON	TOSCAF	UNCEN	Total
**1**	1,702	3,680	45,653	4,045	1,521	2,029	58,630
**2**	34,620	7,581	40,431	2,986	8,326	879	94,823
**3**	2,323	3,923	68	1,168	384	1,440	9,306
**4**	7,870	8,167	0	882	6,769	830	24,518
**5**	2,002	4,567	10,716	360	194	1,232	19,071
**6**	975	1,809	6,373	2,786	128	342	12,413
**7**	7,268	7,563	1,292	2,476	1,246	2,686	22,531
**8**	97,448	5,293	16,334	100	95	14,926	134,196
**9**	10,080	6,074	142	2,195	1,520	1,797	21,808
**10**	86,913	103,232	18,215	6,300	31,503	873	247,036
**11**	4,133	7,278	32,505	1,776	205	2,229	48,126
**12**	5,040	8,021	7	1,371	302	1,199	15,940
**13***	0	0	0	0	0	0	0
**14***	0	0	0	0	0	0	0
**15**	770	2,969	30	223	726	572	5,290
**16**	9,327	6,020	4	1,858	4,886	2,368	24,463
**17**	442	1,825	66,620	792	2,474	155	72,308
**18**	21,263	2,634	1,845	226	736	485	27,189
**19**	6,143	2,900	74	39	282	1,334	10,772
**20**	3,127	4,165	1,153	375	501	735	10,056
**21**	4,081	3,157	0	817	5,163	3,497	16,715
**22**	316	2,477	1	1,280	2,275	50	6,399
**X**	5,485	6,388	9,113	1,802	212	2,236	25,236
**Y**	3,106	3,772	34	482	2,986	1,580	11,960

Table values correspond to read movement graphs shown in Figure 3a and 3b for reads uniquely mapped to unchanged regions of GRCh37 that uniquely map to non-equivalent locations in GRCh38. OFFCEN = movement to centromeric sequence on different chromosome; OFF = moment to non-centromeric sequence on different chromosome; ONCEN = movement to centromeric sequence on same chromosome; ON = movement to non-centromeric sequence on same chromosome; TOSCAF = movement to a non-centromeric unlocalized or unplaced scaffold; UNCEN = movement to a unplaced scaffold containing centromere-associated sequence.
***Read alignments on GRCh38 only involved equivalent locations**

Supplemental Table S5. Counts for NA24143 read movements (multiple GRCh38 mappings)

Chromosome	OFFCEN	OFF	ONCEN	ON	TOSCAF	UNCEN	Total
**1**	47,955	3,037	456,283	11,642	2,361	1,979	523,257
**2**	11,249,872	18,520	192,015	4,399	57,477	9,227	11,531,510
**3**	20,534	7,065	93	544	525	1,680	30,441
**4**	253,222	42,157	0	1,585	25,444	2,136	324,544
**5**	125,035	11,305	10,020	73	395	3,498	150,326
**6**	14,055	2,966	6,534,881	13,959	384	340	6,566,585
**7**	340,127	16,209	496,338	6,861	3,337	8,566	871,438
**8**	128,591	3,240	8,050,389	22	268	24,518	8,207,028
**9**	1,309,293	10,001	472	8,074	4,585	4,853	1,337,278
**10**	4,753,244	216,024	699,930	10,803	87,178	17,673	5,784,852
**11**	160,025	9,077	1,867,094	1,686	1,807	5,096	2,044,785
**12**	73,097	5,603	94	172	539	3,380	82,885
**13***	0	0	0	0	0	0	0
**14***	0	0	0	0	0	0	0
**15**	1,764	6,921	1,446	4,201	3,741	1,191	19,264
**16**	194,760	15,029	24	2,720	18,350	8,512	239,395
**17**	1,302	3,543	244,602	12,501	6,004	374	268,326
**18**	686,094	4,275	59,019	772	1,362	776	752,298
**19**	440,103	7,141	10	96	604	3,838	451,792
**20**	4,986	2,879	3,196	706	1,105	558	13,430
**21**	87,776	8,343	0	13,825	7,983	17,036	134,963
**22**	5,780	1,684	3	1,170	5,962	201	14,800
**X**	159,923	6,422	2,638,769	1,162	518	3,918	2,810,712
**Y**	32,180	7,909	90	1,736	5,019	28,129	75,063

Table values correspond to read movement graphs shown in Supplemental Figure 1b and 1c for reads uniquely mapped to unchanged regions of GRCh37 that map non-uniquely to non-equivalent locations in GRCh38. OFFCEN = movement to centromeric sequence on different chromosome; OFF = moment to non-centromeric sequence on different chromosome; ONCEN = movement to centromeric sequence on same chromosome; ON = movement to non-centromeric sequence on same chromosome; TOSCAF = movement to a non-centromeric unlocalized or unplaced scaffold; UNCEN = movement to a unplaced scaffold containing centromere-associated sequence.

***Read alignments on GRCh38 only involved equivalent locations**

Supplemental Table S6. Summary of de novo assembly methods

Assembly Short Name	GenBank Accession	SRA Sample	Coverage	Chemistry	Assembler	Join Error Threshold	Correction Algorithm
**CHM1_CA_P6**	GCA_001307025.1	SRP044331	61x	P6	CA 8.3rc2	2.5%	falcon_sense
**CHM1_FC_P6**	GCA_001297185.1	SRP044331	61x	P6	FALCON 0.3+	4%	falcon_sense
**CHM13_CA1**	GCA_000983465.1	SRP051383	70x	P5+P6	CA 8.3rc2	5%	falcon_sense
**CHM13_CA2**	GCA_001015355.1	SRP051383	70x	P5+P6	CA 8.3rc2	5%	pbdag-con
**CHM13_CA3**	GCA_000983475.1	SRP051383	70x	P5+P6	CA 8.3rc2	2.5%	falcon_sense
**CHM13_CA4**	GCA_001015385.3	SRP051383	70x	P5+P6	CA 8.3rc2	2.5%	pbdag-con
**CHM13_FC**	GCA_000983455.2	SRP051383	70x	P5+P6	FALCON 0.4	4%	falcon_sense

Supplemental Table S7. Additional de novo assembly statistics

Assembly Short Name	GenBank Accession	Mean Contig Length	Median Contig Length	Max Contig Length	Min Contig Length	Number of Contigs
**CHM1_CA_P6**	GCA_001307025.1	822,968	46,645	109,312,888	2,973	3,641
**CHM1_FC_P6**	GCA_001297185.1	606,109	36,303	99,566,047	3,322	4,850
**CHM13_CA1**	GCA_000983465.1	197,016	16,834	81,522,549	3,057	15,538
**CHM13_CA2**	GCA_001015355.1	271,945	20,645	80,601,297	3,056	11,138
**CHM13_CA3**	GCA_000983475.1	287,288	21,172	34,039,925	3,494	10,430
**CHM13_CA4**	GCA_001015385.3	253,495	21,859	58,473,625	4,833	12,091
**CHM13_FC**	GCA_000983455.2	592,851	31,726	49,307,616	2,781	4,961

Supplemental Table S8: Hybrid Assembly Statistics

	BioNano Assembly # Contigs	Hybrid Assembly # Scaffolds	Hybrid Assembly Scaffold N50 (Mb)	Hybrid Assembly Total Size (Gb)	WGS Assembly Conflicts	BioNano Assembly Conflicts
**CHM1_CA_P6 GCA_001307025.1**	2473	221	40.04	2.82	52	63
**CHM1_FC_P6 GCA_001297185.1**	2473	161	47.60	2.84	45	51
**CHM13_CA1 GCA_000983465.1**	3593	238	29.46	2.81	39	48
**CHM13_CA2 GCA_001015355.1**	3593	229	35.09	2.82	56	83
**CHM13_CA3 GCA_000983475.1**	3593	379	14.80	2.82	29	31
**CHM13_CA4* GCA_001015385.3**	3593	258	27.83	2.82	43	55
**CHM13_FC* GCA_000983455.2**	3593	229	21.66	2.83	63	84

*Conflicts called with different software version than other assemblies (see Methods)

Supplemental Table S9: Structural Variation Calls in WGS-BioNano Assembly Comparison

	SV Type		
**WGS Assembly**	Deletion	Insertion	Inversion
**CHM1_CA_P6 GCA_001307025.1**	25	31	12
**CHM1_FC_P6 GCA_001297185.1**	35	32	7
**CHM13_CA1 GCA_000983465.1**	39	24	6
**CHM13_CA2 GCA_001015355.1**	36	37	11
**CHM13_CA3 GCA_000983475.1**	34	19	42
**CHM13_CA4 GCA_001015385.3***	3	14	7
**CHM13_FC GCA_000983455.2***	15	14	22
*Called with different software version			

Supplemental Table S10. Additional CH17 clone placement statistics

	**CHM1_CA_P6 GCA_001307025.1**	**CHM1_FC_P6 GCA_001297185.1**
**CH17 Clone Ends (n=306,838)**		
**Aligned (Total)**	279,642 (91.14%)	280,674 (91.47%)
**Unique**	272661 (88.86%)	273,064 (88.99%)
**Multiple**	6,981 (2.28%)	7,610 (2.48%)
**Unaligned**	27,196 (8.86%)	26,164 (8.53%)
**CH17 Clones (n=132,368 w/both ends seq'd)**		
**Unique Placement (Concordant)**	96,517	98,188
**Unique Placement (Discordant)**	3,071	3,042
**Length<3*s.d.**	2,479	2,469
**Length>3*s.d.**	41	52
**Incorrect end orientation**	52	51
**Incorrect end orientation and length<3*s.d.**	359	349
**Incorrect end orientation and length>3*s.d.**	140	121
**Multiple Placements**	280	491
**No Placement**	32,219	30,155

Supplemental Table S11. Feature counts for de novo assemblies contributing to FRC curve

Assembly Short Name	COMPR PE*	HIGH COV PE*	HIGH NORM COV PE*	HIGH OUTIE PE*	HIGH SINGLE PE*	HIGH SPAN PE*	LOW COV PE*	LOW NORM COV PE*	STRECH PE*
**CHM1_CA_P6**	4,209	12,214	11,146	55	746	2,482	86,781	88,883	5,570
**CHM1_FC_P6**	6,514	11,937	10,991	60	797	904	81,408	85,064	7,327
**CHM13_CA1**	7,963	13,950	14,085	31,716	0	2,424	58,000	57655	3,370
**CHM13_CA2**	7,413	14,630	14,516	31,078	1	3,287	58,855	58,656	3,672
**CHM13_CA3**	6,230	12,379	12,382	21,491	0	2,347	43,572	43,417	2,924
**CHM13_CA4**	7,764	13,601	13,531	32,993	0	4,057	66,497	65,974	4,006
**CHM13_FC**	8,569	13,592	13,535	7,766	0	1,328	18,617	19,614	2,906

***Features are defined in (Vezzi et al. 2012)**

Supplemental References

Altemose N, Miga KH, Maggioni M, Willard HF. 2014. Genomic characterization of large heterochromatic gaps in the human genome assembly. *PLoS Comput Biol* **10**: e1003628.

Berlin K, Koren S, Chin C-S, Drake JP, Landolin JM, Phillippy AM. 2015. Assembling large genomes with single-molecule sequencing and locality-sensitive hashing. *Nat Biotechnol* **33**: 623–630.

Bradnam KR, Fass JN, Alexandrov A, Baranay P, Bechner M, Birol I, Boisvert S, Chapman JA, Chapuis G, Chikhi R, et al. 2013. Assemblathon 2: evaluating de novo methods of genome assembly in three vertebrate species. *Gigascience* **2**: 10.

Chaisson MJP, Huddleston J, Dennis MY, Sudmant PH, Malig M, Hormozdiari F, Antonacci F, Surti U, Sandstrom R, Boitano M, et al. 2015. Resolving the complexity of the human genome using single-molecule sequencing. *Nature* **517**: 608–611.

Chin C-S, Alexander DH, Marks P, Klammer AA, Drake J, Heiner C, Clum A, Copeland A, Huddleston J, Eichler EE, et al. 2013. Nonhybrid, finished microbial genome assemblies from long-read SMRT sequencing data. *Nat Methods* **10**: 563–569.

Church DM, Schneider VA, Graves T, Auger K, Cunningham F, Bouk N, Chen HC, Agarwala R, McLaren WM, Ritchie GR, et al. 2011. Modernizing reference genome assemblies. *PLoS Biol* **9**: e1001091.

Earl D, Bradnam K, St John J, Darling A, Lin D, Fass J, Yu HOK, Buffalo V, Zerbino DR, Diekhans M, et al. 2011. Assemblathon 1: a competitive assessment of de novo short read assembly methods. *Genome Res* **21**: 2224–2241.

Ewing B, Green P. 1998. Base-calling of automated sequencer traces using phred. II. Error probabilities. *Genome Res* **8**: 186–194.

Garrison E, Marth G. 2012. Haplotype-based variant detection from short-read sequencing. *arXiv [q-bioGN]*. http://arxiv.org/abs/1207.3907.

Genovese G, Handsaker RE, Li H, Altemose N, Lindgren AM, Chambert K, Pasaniuc B, Price AL, Reich D, Morton CC, et al. 2013. Using population admixture to help complete maps of the human genome. *Nat Genet* **45**: 406–14, 414e1–2.

Green RE, Krause J, Briggs AW, Maricic T, Stenzel U, Kircher M, Patterson N, Li H, Zhai W, Fritz MH-Y, et al. 2010. A draft sequence of the Neandertal genome. *Science* **328**: 710–722.

International Human Genome Sequencing Consortium. 2004. Finishing the euchromatic sequence of the human genome. *Nature* **431**: 931–945.

Kitts PA, Church DM, Thibaud-Nissen F, Choi J, Hem V, Sapojnikov V, Smith RG, Tatusova T, Xiang C, Zherikov A, et al. 2016. Assembly: a resource for assembled genomes at NCBI. *Nucleic Acids Res* **44**: D73–80.

Li H. 2013. Aligning sequence reads, clone sequences and assembly contigs with BWA-MEM. *arXiv [q-bioGN]*. http://arxiv.org/abs/1303.3997.

Li H. 2015. FermiKit: assembly-based variant calling for Illumina resequencing data. *Bioinformatics* **31**: 3694–3696.

Pendleton M, Sebra R, Pang AWC, Ummat A, Franzen O, Rausch T, Stütz AM, Stedman W, Anantharaman T, Hastie A, et al. 2015. Assembly and diploid architecture of an individual human genome via single-molecule technologies. *Nat Methods* **12**: 780–786.

Schneider VA, Chen HC, Clausen C, Meric PA, Zhou Z, Bouk N, Husain N, Maglott DR, Church DM. 2013. Clone DB: an integrated NCBI resource for clone-associated data. Nucleic Acids Res 41: D1070–8.

Shi L, Guo Y, Dong C, Huddleston J, Yang H, Han X, Fu A, Li Q, Li N, Gong S, et al. 2016. Long-read sequencing and de novo assembly of a Chinese genome. *Nat Commun* **7**: 12065.

Simpson JT, Durbin R. 2012. Efficient de novo assembly of large genomes using compressed data structures. *Genome Res* **22**: 549–556.

Steinberg KM, Schneider VA, Graves-Lindsay TA, Fulton RS, Agarwala R, Huddleston J, Shiryev SA, Morgulis A, Surti U, Warren WC, et al. 2014. Single haplotype assembly of the human genome from a hydatidiform mole. *Genome Res* **24**: 2066–2076.

Vezzi F, Narzisi G, Mishra B. 2012. Reevaluating assembly evaluations with feature response curves: GAGE and assemblathons. *PLoS One* **7**: e52210.
